# The co-occurrence of mtDNA mutations on different oxidative phosphorylation subunits, not detected by haplogroup analysis, affects human longevity and is population specific

**DOI:** 10.1111/acel.12186

**Published:** 2013-12-17

**Authors:** Nicola Raule, Federica Sevini, Shengting Li, Annalaura Barbieri, Federica Tallaro, Laura Lomartire, Dario Vianello, Alberto Montesanto, Jukka S Moilanen, Vladyslav Bezrukov, Hélène Blanché, Antti Hervonen, Kaare Christensen, Luca Deiana, Efstathios S Gonos, Tom B L Kirkwood, Peter Kristensen, Alberta Leon, Pier Giuseppe Pelicci, Michel Poulain, Irene M Rea, Josè Remacle, Jean Marie Robine, Stefan Schreiber, Ewa Sikora, Peternella Eline Slagboom, Liana Spazzafumo, Maria Antonietta Stazi, Olivier Toussaint, James W Vaupel, Giuseppina Rose, Kari Majamaa, Markus Perola, Thomas E Johnson, Lars Bolund, Huanming Yang, Giuseppe Passarino, Claudio Franceschi

**Affiliations:** 1 BioPhysics and Biocomplexity and Department of Experimental Pathology, C.I. G. Interdepartmental Centre L. Galvani for Integrated Studies on Bioinformatics, University of BolognaBologna, 40126, Italy; 2 BGI Shenzhen, Shenzhen, 518083, China; 3 Department of Cell Biology, University of CalabriaRende, 87036, Italy; 4 Institute of Clinical Medicine, University of Oulu, Oulu University Hospital and MRC OuluOulu, 90014, Finland; 5 Institute of GerontologyKiev, 252114, Ukraine; 6 Centre Polymorphisme Humaine, Fondation Jean DaussetParis, 75010, France; 7 University of TampereTampere, 33014, Finland; 8 Institute of Public Health, University of Southern DenmarkOdense, 5230, Denmark; 9 University of SassariSassari, 07100, Italy; 10 National Hellenic Research FoundationAthens, 116 35, Greece; 11 School of Clinical Medical Sciences, Gerontology “Henry Wellcome”, University of Newcastle upon TyneNewcastle upon Tyne, NE1 3BZ, UK; 12 University of AarhusAarhus, 8000, Denmark; 13 Research & Innovation Soc.Coop. a r.l.Padova, 35127, Italy; 14 IFOM—Fondazione Istituto FIRC di Oncologia MolecolareMilano, 20139, Italy; 15 Research Centre of Demographic Management for Public Administrations, UCL—GéDAPLouvain-la-Neuve, 1348, Belgium; 16 The Queen’s University BelfastBelfast, BT7 1NN, UK; 17 Eppendorf Array Technologies, SA—EAT Research and DevelopmentNamur, 5000, Belgium; 18 University of Montpellier, Val d’Aurelle Cancer Research CenterMontpellier, 34090, France; 19 Kiel Center for Functional Genomics, University Hospital Schleswig HolsteinKiel, 24105, Germany; 20 Nencki Institute of Experimental Biology, Polish Academy of SciencesWarsaw, 00-679, Poland; 21 Leiden University Medical CentreLeiden, 2333 ZA, the Netherlands; 22 INRCA—Italian National Research Centre on AgingAncona, 60127, Italy; 23 Istituto Superiore di SanitàRome, 00161, Italy; 24 Facultés Universitaire Notre Dame de la PaixNamur, 5000, Belgium; 25 Max Planck Institute for Demographic ResearchRostock, 18057, Germany; 26 National Public Health InstituteHelsinki, 00260, Finland; 27 Institute for Behavioral Genetics, University of Colorado BoulderBoulder, CO, 80309, USA

**Keywords:** genetics of longevity, longevity, mitochondrial DNA, mtDNA sequencing, oxidative phosphorylation

## Abstract

To re-examine the correlation between mtDNA variability and longevity, we examined mtDNAs from samples obtained from over 2200 ultranonagenarians (and an equal number of controls) collected within the framework of the GEHA EU project. The samples were categorized by high-resolution classification, while about 1300 mtDNA molecules (650 ultranonagenarians and an equal number of controls) were completely sequenced. Sequences, unlike standard haplogroup analysis, made possible to evaluate for the first time the cumulative effects of specific, concomitant mtDNA mutations, including those that *per se* have a low, or very low, impact. In particular, the analysis of the mutations occurring in different OXPHOS complex showed a complex scenario with a different mutation burden in 90+ subjects with respect to controls. These findings suggested that mutations in subunits of the OXPHOS complex I had a beneficial effect on longevity, while the simultaneous presence of mutations in complex I and III (which also occurs in J subhaplogroups involved in LHON) and in complex I and V seemed to be detrimental, likely explaining previous contradictory results. On the whole, our study, which goes beyond haplogroup analysis, suggests that mitochondrial DNA variation does affect human longevity, but its effect is heavily influenced by the interaction between mutations concomitantly occurring on different mtDNA genes.

## Introduction

Research on mitochondrial biology has underlined the central role played by the mitochondrion in a number of complex traits, including aging and metabolic and degenerative diseases. Mitochondria, due to their central position between energy uptake and energy production, are involved in a number of cellular processes such as heat production, apoptosis regulation, and cellular differentiation. In addition, mitochondria are responsible for the production and the regulation of the most important by-product of the cellular metabolism, reactive oxygen species (ROS), which are widely considered to be one of the main causes of aging (for a review see Passarino *et al*., 2010; Wallace, 2010). Most of the proteins determining the biology of mitochondria are encoded by the nuclear genome, but some important subunits of the oxidative phosphorilation (OXPHOS) are encoded by the mitochondrial DNA (mtDNA). Thus, many studies have addressed the effects of nuclear and mitochondrial DNA mutations (and of the interaction between the two genomes) on mitochondrial functioning and its effect on aging in model organisms and in humans (for a review, see Lagouge & Larsson, 2013; Bratic & Larsson, 2013; Greaves & Turnbull, 2009). In particular, many studies on humans have addressed the role played by the common variability of mitochondrial DNA in modulating the processes influenced by mitochondrial activity, and their effects on degenerative diseases and aging (Brown *et al*., 1997; Ivanova & Lepage, 1998; De Benedictis *et al*., 1999; Ruiz-Pesini *et al*., 2000; Ross *et al*., 2001; Rose *et al*., 2002; Niemi *et al*., 2003; van der Walt *et al*., 2003; Wallace, 2005; Montiel-Sosa *et al*., 2006; Santoro *et al*., 2006). These studies have been carried out mainly by taking advantage of the haplogroup classification of mtDNA molecules. Using this approach, we previously reported a beneficial effect of haplogroup J, present across Europe and the Near East, on longevity, being more frequent in centenarians than in ethnically matched younger controls in northern Italy (De Benedictis *et al*., 1999), Ireland (Ross *et al*., 2001), and Finland (Niemi *et al*., 2003). Intriguingly, haplogroup J was also reported to be strongly associated with LHON (Leber’s hereditary optic neuropathy), increasing the penetrance of specific mutations that are significantly less harmful when occurring in mtDNA molecules of different haplogroups (Brown *et al*., 1997; Torroni *et al*., 1997; Man *et al*., 2004). Similarly, the Asian haplogroup D (characterized by mutations affecting complex I, such as haplogroup J) has been found to be overrepresented in Japanese centenarians (Tanaka *et al*., 1998, 2000). Accordingly, it was proposed that the mutations defining the J haplogroup (falling in the protein subunits of OXPHOS complex I) decrease OXPHOS activity, putting the cell in a vulnerable situation where a further mutation (such as in LHON) would be more likely to be harmful. However, if the nuclear response stimulated by higher ROS production is adequate, the low OXPHOS activity may become advantageous and lead to lower ROS levels, resulting in more healthy aging (Rose *et al*., 2001). Consistent with this hypothesis, cells with mtDNA molecules belonging to haplogroup J were recently found to produce less ATP and less ROS than cells with different mtDNA molecules (Bellizzi *et al*., 2012). However, some studies have failed to find any association between mtDNA and longevity (Dato *et al*., 2004; Pinós *et al*., 2012; Collerton *et al*., 2013), suggesting that the possible effect of mtDNA on longevity might be population specific, but also that such an effect might be minimal, and require a very large number of samples to reach statistical significance. In addition, it is possible that the analysis of mitochondrial DNA haplogroups may not be a suitable approach to uncover the effects of mtDNA given that different sporadic mutations suggested to be important for human longevity (see Beekman *et al*., 2013) fall along mtDNA molecules but are not associated with any of the haplogroups.

Here, we present the results of a very large study, where over 2200 subjects older than 90 years, recruited within the framework of the Genetics of Healthy Ageing (GEHA) EU project (Skytthe *et al*., 2011) and coming from most of the European populations, were compared with a similar number of younger controls matched for sex and geographical origins. In this study, we also availed of the complete sequencing of a subgroup of GEHA samples [650 ultra nonagenarians (90+) and a comparable number of controls, coming from Denmark, Finland, southern Italy and Greece], to determine whether recurrent or sporadic mutations accumulated in specific genes not detected by haplogroup analysis, may influence longevity.

## Results

### Haplogroup analysis

The frequencies of mtDNA haplogroups in 90+ and controls are summarized in Table 1 and, separately for men and women, in Table S1. We observed that in females, the haplogroups H2 and T2 are more frequent in the 90+ group than in controls (*P* = 0.002 and 0.02, respectively). On the other hand, among male controls, the frequency of haplogroups H1 and J2 was higher than in the 90+ group (*P* = 0.023 and 0.004, respectively). No association was detectable when multiple test corrections were used.

**Table 1:** Distribution of mtDNA haplogroups in the Genetics of Healthy Ageing (GEHA) samples

	Controls (*n* = 2153)		90+ (*n* = 2086)		
Subhaplogroups	*N*	%	*N*	%	*P*-value
HV*	1042	48.4	1052	50.43	0.187
HV0*	68	3.16	83	3.98	0.159
H*	924	42.92	927	44.44	0.322
H1	325	15.1	292	14	0.317
H2	23	1.07	42	2.01	0.013
H3	85	3.95	71	3.4	0.370
H5	63	2.93	68	3.26	0.536
H6	47	2.18	52	2.49	0.542
I	44	2.04	46	2.21	0.750
J	210	9.75	183	8.5	0.289
J1	153	7.11	144	6.9	0.810
J2	57	2.65	39	1.87	0.099
K	161	7.48	137	3.98	0.254
K1	140	6.5	117	5.61	0.247
T	211	9.8	223	10.69	0.362
T1	53	2.46	43	2.06	0.410
T2	152	7.06	174	8.34	0.120
U	313	14.54	272	13.04	0.167
U2	39	1.81	38	1.82	1.000
U4	48	2.23	34	1.63	0.181
U5a	109	5.06	90	4.31	0.276
U5b	56	2.6	51	2.44	0.770
W	39	1.81	51	2.44	0.166
X	41	1.9	42	2.01	0.825
OTHER	92	5.53	80	5.08	0.484

* is part of the internationally used designation of these groups of haplotypes.

Haplogroups and subhaplogroups with frequencies higher than 1.5% are reported. OTHER includes N1a, N1b, N1c, N9a, A4, D5, C1d, M1, L1b1.

### Sequence analysis

The mtDNA sequences have been deposited in GenBank, with accession numbers JX152783 – JX154074.

To analyze the mtDNA sequences, we first compared all complete sequences with the reference sequence (rCRS). A summary of all identified mutations is shown in Table S2. The analysis of single mutations did not highlight any specific mutation that could consistently account for an advantage in longevity.

Thus, we analyzed the possibility that nonsynonymous mutations may alter specific genes and affect the chances of attaining extreme longevity. We analyzed mtDNA sequences of ultranonagenarians and controls to find an aggregate mtDNA variation associated with longevity. To avoid false-positive results as much as possible, the analysis of mtDNA sequences was carried out using a gene-based kernel approach (Wu *et al*., 2011), adjusted for genetic ancestry by means of principal component analysis according to Biffi *et al*. (2010) and corrected for multiple testing (number of ‘genes’ analyzed). In our case, we considered a ‘gene’ as all the subunits encoded by mtDNA and belonging to a single complex of OXPHOS (ND 1-6 and ND4L are in complex I, Cyt b in complex III, COI-III in complex IV, ATPase 6 and 8 in complex V). Table 2 reports the results of the gene-based analysis, while Fig. S1 shows the results of the underlying principal component analysis. Table 2 clearly shows that nonsynonymous mutations falling within Complex I, III and V are associated with longevity in our sample. By contrast, there was no correlation between synonymous mutations falling in different subunits of OXPHOS encoded by mitochondrial DNA. No significant correlation was observed for either rRNA genes or tRNA genes.

**Table 2:** Results of sequence analysis according to sequence kernel association test (SKAT) to find aggregate gene-based associations for human longevity using the 1840 mtDNA SNPs

			Nonsynonymous mutations		Synonymous mutations	
	Number of SNPs	*P*-value	Number of SNPs	*P*-Value	Number of SNPs	*P*-value
Complex I	712		200	0.02648710	512	0.39526309
Complex III	136		54	0.03876515	82	0.17760404
Complex IV	321*		85	0.51612941	235	0.36290044
Complex V	134		74	0.00057112	60	0.14215469
rRNA	154	0.43332840				
tRNA	100	0.26393589				
Control region	267	0.10580026				

*P*-values were obtained, by bootstrap analysis (10 000 bootstrap), using the SKAT algorithm after adjusting for the first four principal components.

* One position is noncoding (position 8269, in COII).

**Figure 1:**
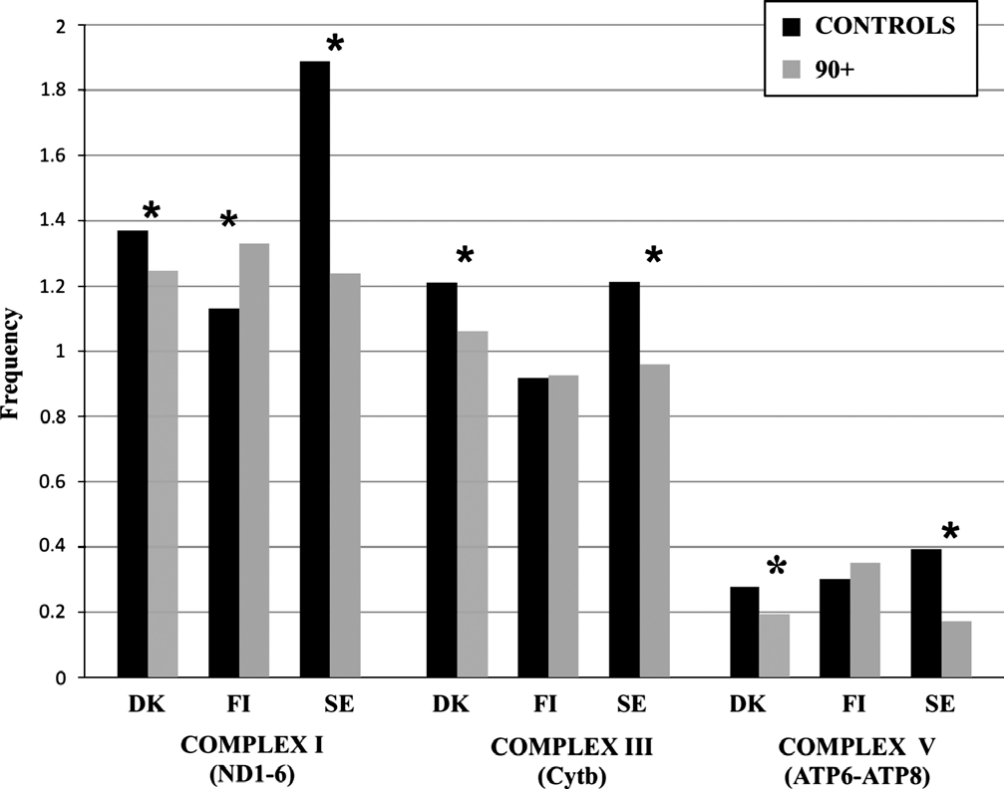
Nonsynonymous mutation frequencies in 90+ and controls in mtDNA genes of OXPHOS complexes I, III and V, which showed a significant association with longevity after sequence analysis for pooled associations (Table 2). * indicates statistical significant differences between 90+ and controls. Frequency (y-axis) indicates the average number of mutation per subject every 100 bp of the relevant mtDNA region. DK, FI, and SE stand for Denmark, Finland, and South Europe populations, respectively.

To better understand the results and, in particular, to determine whether mutations falling in different complexes have a positive or negative effect, we compared 90+ and controls in different populations for nonsynonymous mutations in genes belonging to the mitochondrial complex that was significantly associated with longevity upon the gene-based sequence analysis above (Fig. 1). When we considered the subunits of complex I, all three populations showed a difference between 90+ and controls. However, the frequency of nonsynonymous mutations was higher in controls than in the 90+ belonging to Danish and southern European populations (respectively, *P* = 0.03 in Denmark and *P* << 0.001 in South Europe), while in the Finnish population, we observed the opposite trend (*P* = 0.02).

For complex III and V, Danish and southern European populations showed a higher frequency of mutations in controls than in the 90+ group (*P* = 0.005 in Denmark and *P* = 0.048 in South Europe) (*P* = 0.0011 and *P* = 0.0023, respectively), while in the Finnish population, the mutation frequency did not differ between the 90+ and controls for both complexes.

Given the absolute linkage among mtDNA mutations, we investigated whether the different results obtained in our samples (with Finns showing a different trend with respect to other samples) could be due to a different distribution of the simultaneous mutations in subunits of different complexes. As most of the samples showed at least one mutation in both complexes I and III, for each sample, we counted the subjects where we observed two or more mutations in the subunits of both complex I and complex III (Table 3a). Indeed, we observed that, when controls are considered, both Danes and southern Europeans show a higher frequency of the simultaneous presence of two mutations in complex I and III than Finns (*P* = 0.04 and *P* = 0.006, respectively). The same result was obtained when complex I and V were considered (Table 3b). In both cases, the number of subjects with two or more mutations in complex I and III or in complex I and V was higher in controls than in ultranonagenarians (*P* = 0.03 and *P* = 0.02, respectively).

**Table 3:** (a) Samples with 2 or more mutations in both complex I and III; (b) samples with 2 or more mutations in both complex I and V

Population (n. total sample)	n. sample	%
(a)		
Denmark controls (429)	131	30.53
inland controls (166)	32	21.91
South Europe controls (71)	28	39.43
Total controls (666)	191	28.67*
Denmark 90+ (423)	117	27.66
Finland 90+ (148)	23	15.54
South Europe 90+ (75)	22	18.66
Total 90+(646)	162	25.07*
(b)		
Denmark controls (429)	55	12.82
Finland controls (166)	17	10.24
South Europe controls (71)	13	18.31
Total Controls (666)	85	12.76†
Denmark 90+ (423)	39	9.22
Finland 90+ (148)	11	7.43
South Europe 90+ (75)	7	9.33
Total 90+(646)	57	8.82†

* The co-occurrence of two or more mutations in complex I and III is more frequent in controls than in 90+ (*P* = 0.03).

† The co-occurrence of two or more mutations in complex I and V is more frequent in controls than in 90+ (*P* = 0.02).

## Discussion

Previous analyses on the correlation between mitochondrial DNA variability and longevity in humans have produced interesting but contradictory results (Passarino *et al*., 2010 and references therein). The large GEHA sampling of European ultranonagenarians with sex- and geographically matched controls, and the complete sequencing of a great number of mtDNA molecules allowed us to shed light on this contradictory scenario. The unprecedented number of samples collected and analyzed makes these results very relevant in the longevity and mtDNA fields. The results are reinforced by the specific attention to the recruitment procedure aimed at minimizing population sampling bias stipulated by the GEHA project (see Experimental procedures).On the other hand, it must be acknowledged that cases and controls come from different birth cohorts, as it happens for studies on longevity, and this may represent a limitation.

The analysis of haplogroups showed that haplogroups H2 and T2 are associated with longevity in females. Haplogroup J2 was also associated with longevity in males, showing an opposite trend with respect to previous studies (Niemi *et al*., 2003; Domínguez-Garrido *et al*., 2009). In fact, J2 turned out to be less frequent in 90+ than in controls. Although none of these associations held after multiple tests adjustment, it is interesting to note that the haplogroups H2, T (including T2) and J (including J2) are characterized by nonsynonymous mutations in subunits of OXPHOS complex I, which, as mentioned above, has previously, although not consistently, been suggested to be associated with longevity.

Although the analysis of mtDNA haplogroups provided some interesting data, it proved once again to be unable to fully shed light on the correlation between mtDNA variability and longevity. The analysis of sequencing data showed that the pooled nonsynonymous variability of each of OXPHOS complexes I, III and V is associated with longevity. However, complex I exhibited higher mutation frequency in the 90+ group than in controls in Finns (suggesting that mutations in these subunits may be beneficial), but it showed more mutations in controls than in 90+ in Danish and in southern Europeans (suggesting that mutations in the subunits of complex I may be detrimental). On the other hand, also for complexes III and V, Danes and southern Europeans had a similar trend with more nonsynonymous mutations in controls than in ultranonagenarians, while Finns showed no differences between ultranonagenarians and controls for both complexes. The analysis of the simultaneous presence of mutations on both complexes I and III and in both complexes I and V suggests that the presence of mutation on complex I may be beneficial for longevity, while the co-occurrence of mutations on both complexes I and III or on both I and V might lower the individual’s chances for longevity. It might be worth mentioning in this context that studies on the molecular basis of LHON showed that the penetrance of LHON mutations is greatly increased by the concomitant occurrence of mutations on complexes I and III defining specific J subhaplogroups, such as J1c and J2b (Carelli *et al*., 2006; Achilli *et al*., 2012). Accordingly, this result might explain why the J haplogroup is associated with longevity in some populations but not in others. In populations where J subhaplogroups with additional mutations in complex III (or complex V) are more frequent, we may expect to find no association between J and longevity, similarly to what has been reported regarding the absence of correlation between haplogroup J and LHON in populations where these subhaplogroups are not present (Carelli *et al*., 2006). Indeed previous analyses had shown that haplogroup J is associated with longevity in Finns but not in southern Italians (De Benedictis *et al*., 1999; Niemi *et al*., 2003; Dato *et al*., 2004). On the basis of our results, we can hypothesize that mutations in complex I may decrease the rate of OXPHOS, possibly by slowing the initiation rate of the electron transport chain, thus leading to a lower production of both ATP and ROS, as suggested in an *in vitro* study by Bellizzi *et al*. (2012). In contrast, the impairment of supercomplex I–III (or of complexes I and V) can affect the activity of OXPHOS by decreasing (or, at worse, blocking) the initiated electron transport chain, leading to higher ROS production and/or hyperpolarization of the mitochondrial membrane (Wallace, 2005). This hypothesis takes into account available data but will certainly need to be studied *in vivo* using appropriate models with specific mutations. In fact, our results are based on the different genetic burden of NS mutations in 90+ participants when compared with controls, but they are limited to explain epistatic interactions and the real effect of the mutations. Future work will need to better characterize the molecular effects of single mutations. Moreover, it will be important also to better analyze the interaction between these mutations and mutations in the regulatory region affecting mtDNA expression, as the 295C/T transition which was showed to affect mitochondrial transcription and copy number in cybrid models and is typical of haplogroup J (Suissa *et al*., 2009).

It is also worth mentioning that the availability of complete sequences allowed us to evaluate the frequency of mutations previously reported to be associated with degenerative diseases in 90+ and controls (Table S3). Unexpectedly, such mutations are present in the 90+ group sometimes at higher frequencies than in controls. For instance, the 4336T>C mutation in the tRNA^gln^ has been found to be more frequent in patients with AD than in controls by a number of studies between 1993 and 2010 (Shoffner *et al*., 1993; Hutchin & Cortopassi, 1995; Brown *et al*., 1996; Santoro *et al*., 2010). We found this mutation to be twice as frequent in 90+ than in controls. Similarly, many other supposed deleterious mutations have been found to be quite frequent in 90+. These results support the idea that certain mtDNA-inherited mutations could contribute to longevity or disease according to population- and individual-specific genetic backgrounds (nuclear genome and combination of mtDNA mutations), as well as stochastic events (Rose *et al*., 2001; Moilanen *et al*., 2003; Wallace, 2010).

In conclusion, we postulate that particular rare mtDNA mutations, present only in specific populations, might be beneficial (or detrimental) for longevity and may explain part of the genetic component of longevity in that population, similarly to what has been suggested for private nuclear DNA polymorphisms (Beekman *et al*., 2013). On the whole, our study has allowed the reappraisal of the correlation between mtDNA variability and longevity. We show that complete mtDNA sequencing and analysis of its whole variability can highlight correlations between mtDNA variability and a specific phenotype, such as longevity which escapes standard haplogroup analysis. In fact, complete sequence analysis allows the appreciation of the effects of rare or repeated mutations unaccounted for in haplogroup analysis (Rollins *et al*., 2009). In addition, we have better highlighted the beneficial effects on longevity of mutations in the subunits of OXPHOS complex I and the detrimental effects of mutations in subunits of complex III or V (also in presence of mutations in complex I). It is important to underline that the very small effect of these mutations on longevity requires a very large sample size to be significantly highlighted.

Finally, we have also shown that mtDNA mutations, previously found to be associated with a number of degenerative disorders, are present in ultranonagenarians and in some cases even at higher frequencies than in controls. Again, this suggests that mtDNA mutations can be either positive or negative according to other factors, most likely including environmental factors as well as other mitochondrial and nuclear genomic background.

## Experimental procedures

### Sampling

Samples were collected within the framework of the GEHA research project (2004–2010) in 11 European Countries. Each institution providing blood samples received the approval from its own ethical committee, and all the recruited subjects provided written informed consent for the use of their phenotypic and genetic data in studies on human aging (Skytthe *et al*., 2011).

Within GEHA, families with two or more siblings 90 years old or older were identified with different strategies depending on the available information on the population. Once identified, individuals were contacted and an appointment was made to visit the individual in his or her usual place of residence. During the visit, a structured interview was conducted based on a standardized questionnaire, and blood samples consisting of three 7.5 mL EDTA tubes of blood were collected from each participant. For this study, we used only the older brother of each sibship.

The following inclusion criteria were applied to the recruitment of younger controls: (i) the participant should be between 50 and 75 years of age; (ii) he or she should be genetically unrelated to the old siblings to whom he/she was assigned as control; (iii) he or she should be of the same ethnicity and geographical background as the old siblings; (iv) whenever possible, he or she should be of the same gender as the oldest sibling in the sib pair or group. In cases where it was possible to contact the spouse of a child of an old sibling, he/she was asked to participate. Alternatively, control volunteers were recruited from the same geographical area as the ultranonagenarians (further details on the sampling can be found on Skytthe *et al*., 2011).

DNA was recovered from fresh blood by automated and standardized protocol to guarantee quality and concentration uniformity among samples. The GEHA Partner in charge of the DNA extraction (KTL – Helsinki, Finland) provided 4 μg of genomic DNA for mtDNA variability analysis.

A total of 4239 samples were available for mitochondrial haplogroup classification and for exploring the possible association between mtDNA-inherited sequence variation and longevity. Samples were distributed among ultranonagenarians (90+) and younger controls as reported in Table S4 and had the features reported in Table S5 (Supporting Information).

### mtDNA Sequence Variation Screening

From the 4239 samples available, 1292 (646 ultranonagenarians and 646 controls) were selected for complete sequencing to analyze the differences between northern and southern European populations in the whole DNA molecule. For the remaining 2947 samples (1449 ultranonagenarians and 1498 controls), the mtDNA subhaplogroup was determined to verify possible haplogroup association with longevity and to compare haplogroup distribution among different European countries.

Complete sequencing was performed using two different protocols previously compared for the reliability of results by parallel testing of samples. A total of 125 sequences (109 Calabrians and 16 Greeks) were obtained using the MitoALL Resequencing kit (Applera, Foster City, CA, USA) introducing four alternative primer pairs to improve the amplification rate (Table S6). Each amplification step was followed by purification with EXOSAPit (U.S. Biochemical, Cleveland, Ohio). The sequencing reaction was conducted using the BigDye Kit, version 3.1 (Applera), and M13 universal primers (forward and reverse), followed by purification of the sequences by ethanol precipitation. Electropherograms were analyzed with Seqscape, version 2.5 software (Applera), that allows the assembly of all 46 fragments belonging to the sample and to align and compare the obtained consensus sequence with the revised Cambridge Reference Sequence (rCRS, NCBI: NC_012920.1 gi:251831 106) (Andrews *et al*., 1999). Two independent operators manually verified sequences for phantom mutations by reads of both strands. The remaining samples were sequenced in Beijing Genomics Institute (BGI) as previously described (Wang *et al*., 2008).

The definition of the mtDNA subhaplogroups in the remaining 2947 samples was conducted by resequencing the D-loop region from nucleotide position (np) 16024 to np 576 followed by RFLP analysis in specific coding region traits as previously described (Torroni *et al*., 1996).

For the haplogroup and subhaplogroup assignment, we followed the Phylotree nomenclature (www.phylotree.org) (van Oven & Kayser, 2009). Considering the number of samples to classify, we developed a custom tree search algorithm that, coupled with a highly-efficient SNP discovery pipeline, is able to define the haplogroup that better matches the sample mutational motifs, starting from the raw sequence in FastA format (Vianello *et al*., 2013).

### Statistical analysis

r statistical software was used for all statistical analyses. mtDNA subhaplogroups and relevant frequencies were stratified by gender and were compared between 90+ subjects and their ethnically matched controls using the chi-squared test with Pearson correction or Fisher’s exact test. The comparison of frequencies in 90+ and younger controls of each mtDNA subhaplogroup was computed by applying Pearson’s chi-squared test with Yates’ continuity correction. Tests for statistical significance were two-sided with α = 0.05.

Principal component analysis with mitochondrial SNPs passing quality control (MAF > 0.01), was used to control for hidden population substructure in the following association analyses (Biffi *et al*., 2010). This method has been shown to outperform haplogroup-stratified or haplogroup-adjusted association analyses with no loss in power for the detection of true associations. Principal component analysis was performed using the SNPRelate R package (Zheng *et al*., 2012) using only individuals of European ancestry.

Rare-Variant association testing for sequencing data was carried out to detect rare mtDNA variants involved in human longevity. For this purpose, the SKAT algorithm was used: a supervised and flexible regression method to test for association between rare variants in a gene or genetic region with a continuous/dichotomous trait. This algorithm was implemented in the SKAT package of R. Compared with other gene-based approach, SKAT is able to deal with variants that have different direction and magnitude of effects, allows for covariate adjustment, and also avoids arbitrary selection of threshold in burden test (Wu *et al*., 2011). Gene-based analysis was adjusted for the first four PCs derived from the principal component analysis previously described. To compute the SKAT *P*-values, we used a resampling procedure based on 10 000 bootstraps. The function used to measures the genetic similarity between two random subjects i and i’ in the analyzed region via the *P* selected markers was the weighted linear kernel function. As suggested by Wu *et al*. (2011), the diagonal weight matrix was chosen using the beta distribution density function with parameters a1 = 1 and a2 = 25 evaluated at the sample minor-allele frequency or MAF (default option of SKAT package of R).

## Supporting Information

**Fig. S1** Scatterplot of the first two principal component scores based on the 229 SNPs (MAF > 0.01) identified in the 1291 mtDNA analyzed sequences.

**Table S1.** a and b. Haplogroup distribution in males and females.

**Table S2.** List of all the mutations identified in our sample by the complete mtDNA sequencing.

**Table S3.** mtDNA mutations found in our study and previously associated with diseases. **Table S4.** Distribution of 90+ and controls from different centers participating in the GEHA project. **Table S5.** Sample distribution and general characteristics of the study participants. **Table S6.** Modified primer pairs for complete resequencing.
