# Erratum

**DOI:** 10.1111/acel.12377

**Published:** 2015-09-03

In Raule *et al*. (2014), there was an error in the listed affiliation for author, Pier Giuseppe Pelicci. The correct affiliations are shown below:

^1^Department of Experimental Oncology, European Institute of Oncology, Milan, Italy

^2^Dipartimento di Scienze della Salute, Universita’ degli Studi di Milano, Italy

The publishers would like to apologise for this error.

## References

[b1] Raule N, Sevini F, Li S, Barbieri A, Tallaro F, Lomartire L, Vianello D, Montesanto A, Moilanen JS, Bezrukov V, Blanché H, Hervonen A, Christensen K, Deiana L, Gonos ES, Kirkwood TBL, Kristensen P, Leon A, Pelicci PG, Poulain M, Rea IM, Remacle J, Robine JM, Schreiber S, Sikora E, Eline SlagboomP, Spazzafumo L, Antonietta StaziM, Toussaint O, Vaupel JW, Rose G, Majamaa K, Perola M, Johnson TE, Bolund L, Yang H, Passarino G, Franceschi C (2014). The co-occurrence of mtDNA mutations on different oxidative phosphorylation subunits, not detected by haplogroup analysis, affects human longevity and is population specific. Aging Cell.

