# Supplementary material for: The co-occurrence of mtDNA mutations on different oxidative phosphorylation subunits, not detected by haplogroup analysis, affects human longevity and is population specific
Source: Aging Cell. 2013 Dec 17;13(3):401–7. doi: 10.1111/acel.12186 (PMC4326891; doi:10.1111/acel.12186)
Supplement: Supplementary file 1 — Fig. S1 Scatterplot of the first two principal component scores based on the 229 SNPs (MAF > 0.01) identified in the 1291 mtDNA analyzed sequences. [file acel0013-0401-sd1.pdf]

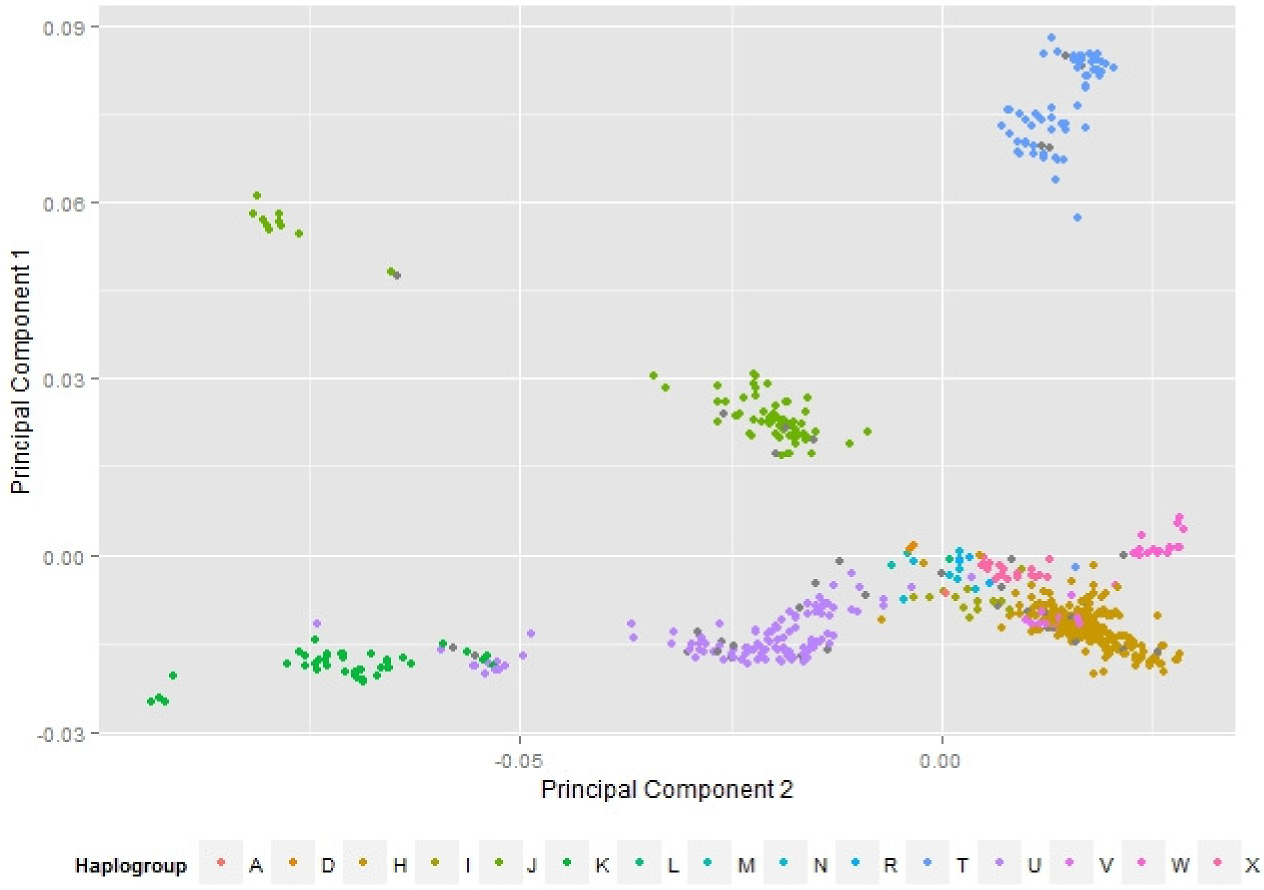

Fig.1S. Scatterplot of the first two principal component scores based on the 229 SNPs (MAF>0.01) identified in the 1291 mtDNA analyzed sequences. Colored dots convey information about haplogroup assignment for each individual. As expected, PCA recapitulated the population structure and reflected haplogroup assignment (Biffi et al. 2010). For the SKAT analysis also the third and fourth Principal Components were used.
