# Supplementary material for: The co-occurrence of mtDNA mutations on different oxidative phosphorylation subunits, not detected by haplogroup analysis, affects human longevity and is population specific
Source: Aging Cell. 2013 Dec 17;13(3):401–7. doi: 10.1111/acel.12186 (PMC4326891; doi:10.1111/acel.12186)
Supplement: Supplementary file 2 — Table S1. a and b. Haplogroup distribution in males and females. [file acel0013-0401-sd2.pdf]

Table 1S a. Haplogroup distribution in males. SE= Standard Error. Sub-haplogroups with frequencies lower than 1.5% were grouped. HV0* includes HV0a, OTHER includes K, K2, T, R0, R0a, R1, R2, HV1, HV2, N1a, N1b, N1c,N9a, A4, D5, C1d, M1, L1b1, with frequencies lower than 1.5%.

	**Males (N=1327)**				
	**Controls (N= 699)**		**90+ (N=628)**		**P-value**
Sub-haplogroups	**N**	**%**	**N**	**%**	
**HV***	347	49.6	318	50.6	0.447
**HV0***	24	3.4	24	3.8	1.000
**H***	306	43.8	280	44.6	0.476
**H1**	108	15.5	75	11.9	0.023
**H2**	11	1.6	10	1.6	1.000
**H3**	16	2.3	18	2.9	0.732
**H5**	20	2.9	24	3.8	0.545
**H6**	16	2.3	19	3	0.612
**I**	13	1.9	7	1.1	0.263
**J1**	44	6.3	40	6.4	0.826
**J2**	22	3.1	6	1	0.004
**K1**	46	6.6	47	7.5	0.834
**T1**	11	1.6	11	1.8	1.000
**T2**	54	7.7	46	7.3	0.545
**U**	93	13.3	88	14.0	0.876
**U2**	13	1.9	12	1.9	1.000
**U4**	13	1.9	13	2.1	1.000
**U5a**	30	4.3	24	3.8	0.497
**U5b**	17	2.4	18	2.9	0.866
**W**	16	2.3	13	2.1	0.711
**X**	19	2.7	18	2.9	1.000
**OTHER**	34	4.9	34	5.4	0.903

Table 1S b. Haplogroup distribution in females. SE=Standard Error; Sub-haplogroups with frequencies lower than 1.5% were grouped. HV0* includes HV0a, OTHER includes K, K2, T, R0, R0a, R1, R2, HV1, HV2, N1a, N1b, N1c,N9a, A4, D5, C1d, M1, L1b1, with frequencies lower than 1.5%.

Females (N = 2912)

	**Controls (N= 1454)**		**90+ (N= 1458)**		**p-value**
	**N**	**%**	**N**	**%**	
**HV***	695	47.8	734	50.3	0.047
**HV0***	44	3	59	4	0.110
**H***	618	42.5	647	44.4	0.107
**H1**	217	14.9	217	14.9	0.761
**H2**	12	0.8	32	2.2	0.002
**H3**	69	4.7	53	3.6	0.200
**H5**	43	3	44	3	0.829
**H6**	31	2.1	33	2.3	0.708
**I**	31	2.1	39	2.7	0.281
**J1**	109	7.5	104	7.1	0.944
**J2**	35	2.4	33	2.3	1.000
**K1**	94	6.5	70	4.8	0.094
**T1**	42	2.9	32	2.2	0.348
**T2**	98	6.7	128	8.8	0.024
**U**	220	15.1	184	12.6	0.910
**U2**	26	1.8	26	1.8	1.000
**U4**	35	2.4	21	1.4	0.082
**U5a**	79	5.4	66	4.5	0.398
**U5b**	39	2.7	33	2.3	0.635
**W**	23	1.6	38	2.6	0.052
**X**	22	1.5	24	1.6	0.767
**OTHER**	85	5.8	72	4.9	0.416
