# Supplementary material for: The co-occurrence of mtDNA mutations on different oxidative phosphorylation subunits, not detected by haplogroup analysis, affects human longevity and is population specific
Source: Aging Cell. 2013 Dec 17;13(3):401–7. doi: 10.1111/acel.12186 (PMC4326891; doi:10.1111/acel.12186)
Supplement: Supplementary file 3 — Table S2. List of all the mutations identified in our sample by the complete mtDNA sequencing. [file acel0013-0401-sd3.pdf]

Table 2S. List of the SNPs identified in our sample and used for the SKAT analysis (Wu et al.)

SNP	POSITION	A1	F_A	F_U	A2	GENE	Amino.Acid.Change
P9	9	A	0,0016	0,0000	G	Control-Region	-
P16	16	T	0,0000	0,0046	A	Control-Region	-
P55	55	C	0,0016	0,0015	T	Control-Region	-
P57	57	C	0,0093	0,0031	T	Control-Region	-
P58	58	C	0,0016	0,0015	T	Control-Region	-
P59	59	C	0,0031	0,0015	T	Control-Region	-
P61	61	T	0,0031	0,0015	C	Control-Region	-
P62	62	T	0,0016	0,0000	G	Control-Region	-
P63	63	C	0,0016	0,0000	T	Control-Region	-
P66	66	C	0,0016	0,0000	G	Control-Region	-
P73	73	A	0,4845	0,4389	G	Control-Region	-
P93	93	G	0,0202	0,0201	A	Control-Region	-
P94	94	A	0,0047	0,0046	G	Control-Region	-
P95	95	C	0,0016	0,0015	A	Control-Region	-
P108	108	G	0,0000	0,0015	A	Control-Region	-
P114	114	T	0,0062	0,0093	C	Control-Region	-
P119	119	C	0,0000	0,0031	T	Control-Region	-
P121	121	A	0,0000	0,0015	G	Control-Region	-
P127	127	C	0,0000	0,0031	T	Control-Region	-
P131	131	C	0,0031	0,0000	T	Control-Region	-
P132	132	T	0,0000	0,0015	C	Control-Region	-
P143	143	A	0,0093	0,0031	G	Control-Region	-
P146	146	C	0,0854	0,0804	T	Control-Region	-
P150	150	T	0,0868	0,0804	C	Control-Region	-
P151	151	T	0,0078	0,0093	C	Control-Region	-
P152	152	C	0,1726	0,1732	T	Control-Region	-
P153	153	G	0,0155	0,0186	A	Control-Region	-
P181	181	T	0,0016	0,0000	A	Control-Region	-
P182	182	T	0,0016	0,0031	C	Control-Region	-
P183	183	G	0,0031	0,0031	A	Control-Region	-

A1: Minor allele name (whole sample)
F_A: Frequency of allele A1 in cases
F_U: Frequency of allele A1 in controls
A2: Major allele name

P186	186	A	0,0031	0,0031	C	Control-Region	-
P188	188	G	0,0341	0,0340	A	Control-Region	-
P189	189	G	0,0326	0,0340	A	Control-Region	-
P192	192	C	0,0031	0,0015	T	Control-Region	-
P193	193	G	0,0031	0,0015	A	Control-Region	-
P194	194	T	0,0124	0,0062	C	Control-Region	-
P195	195	C	0,1659	0,1778	T	Control-Region	-
P196	196	C	0,0093	0,0015	T	Control-Region	-
P198	198	T	0,0062	0,0062	C	Control-Region	-
P199	199	C	0,0357	0,0278	T	Control-Region	-
P200	200	G	0,0047	0,0093	A	Control-Region	-
P203	203	A	0,0093	0,0015	G	Control-Region	-
P204	204	C	0,0544	0,0433	T	Control-Region	-
P207	207	A	0,0310	0,0278	G	Control-Region	-
P210	210	G	0,0000	0,0015	A	Control-Region	-
P214	214	G	0,0016	0,0015	A	Control-Region	-
P215	215	G	0,0217	0,0247	A	Control-Region	-
P217	217	C	0,0186	0,0170	T	Control-Region	-
P222	222	T	0,0016	0,0000	C	Control-Region	-
P225	225	A	0,0140	0,0201	G	Control-Region	-
P226	226	C	0,0109	0,0062	T	Control-Region	-
P227	227	G	0,0078	0,0062	A	Control-Region	-
P228	228	A	0,0589	0,0773	G	Control-Region	-
P234	234	G	0,0062	0,0062	A	Control-Region	-
P235	235	G	0,0031	0,0077	A	Control-Region	-
P239	239	C	0,0202	0,0309	T	Control-Region	-
P242	242	T	0,0047	0,0062	C	Control-Region	-
P245	245	C	0,0016	0,0000	T	Control-Region	-
P246	246	C	0,0047	0,0015	T	Control-Region	-
P247	247	A	0,0000	0,0046	G	Control-Region	-
P249	249	G	0,0000	0,0015	A	Control-Region	-
P250	250	C	0,0202	0,0124	T	Control-Region	-
P253	253	T	0,0000	0,0015	C	Control-Region	-

P256	256	T	0,0000	0,0031	C	Control-Region	-
P257	257	G	0,0062	0,0062	A	Control-Region	-
P260	260	A	0,0016	0,0000	G	Control-Region	-
P263	263	A	0,0326	0,0371	G	Control-Region	-
P271	271	T	0,0016	0,0000	C	Control-Region	-
P279	279	C	0,0078	0,0062	T	Control-Region	-
P280	280	T	0,0016	0,0000	C	Control-Region	-
P282	282	C	0,0031	0,0031	T	Control-Region	-
P285	285	T	0,0000	0,0031	C	Control-Region	-
P293	293	C	0,0000	0,0031	T	Control-Region	-
P300	300	C	0,0000	0,0031	A	Control-Region	-
P301	301	C	0,0016	0,0000	A	Control-Region	-
P302	302	C	0,0016	0,0015	A	Control-Region	-
P303	303	A	0,0000	0,0015	C	Control-Region	-
P309	309	T	0,0265	0,0278	C	Control-Region	-
P310	310	C	0,0016	0,0016	T	Control-Region	-
P316	316	A	0,0016	0,0000	G	Control-Region	-
P319	319	C	0,0155	0,0186	T	Control-Region	-
P321	321	C	0,0047	0,0000	T	Control-Region	-
P327	327	T	0,0031	0,0000	C	Control-Region	-
P334	334	C	0,0016	0,0015	T	Control-Region	-
P340	340	T	0,0062	0,0046	C	Control-Region	-
P366	366	A	0,0016	0,0000	G	Control-Region	-
P368	368	G	0,0000	0,0015	A	Control-Region	-
P373	373	G	0,0000	0,0015	A	Control-Region	-
P385	385	G	0,0016	0,0015	A	Control-Region	-
P415	415	G	0,0000	0,0015	A	Control-Region	-
P430	430	C	0,0000	0,0015	T	Control-Region	-
P438	438	T	0,0000	0,0015	C	Control-Region	-
P444	444	G	0,0000	0,0015	A	Control-Region	-
P451	451	G	0,0016	0,0000	A	Control-Region	-
P453	453	C	0,0031	0,0031	T	Control-Region	-
P456	456	T	0,0047	0,0046	C	Control-Region	-

P460	460	C	0,0000	0,0015	T	Control-Region	-
P462	462	T	0,0760	0,0804	C	Control-Region	-
P463	463	T	0,0000	0,0015	C	Control-Region	-
P466	466	C	0,0000	0,0015	T	Control-Region	-
P469	469	T	0,0016	0,0000	C	Control-Region	-
P477	477	C	0,0357	0,0433	T	Control-Region	-
P480	480	C	0,0000	0,0015	T	Control-Region	-
P482	482	C	0,0062	0,0186	T	Control-Region	-
P485	485	C	0,0000	0,0046	T	Control-Region	-
P489	489	C	0,1008	0,1190	T	Control-Region	-
P493	493	G	0,0000	0,0015	A	Control-Region	-
P497	497	T	0,0233	0,0232	C	Control-Region	-
P499	499	A	0,0171	0,0232	G	Control-Region	-
P505	505	T	0,0016	0,0000	C	Control-Region	-
P508	508	G	0,0140	0,0108	A	Control-Region	-
P524	524	A	0,0000	0,0017	C	Control-Region	-
P533	533	G	0,0016	0,0062	A	Control-Region	-
P538	538	G	0,0016	0,0015	A	Control-Region	-
P567	567	C	0,0031	0,0046	A	Control-Region	-
P569	569	T	0,0016	0,0015	C	Control-Region	-
P574	574	C	0,0047	0,0000	A	Control-Region	-
P575	575	T	0,0016	0,0015	C	Control-Region	-
P593	593	C	0,0016	0,0031	T	tRNA	-
P629	629	C	0,0016	0,0000	T	tRNA	-
P633	633	T	0,0000	0,0015	A	tRNA	-
P636	636	G	0,0016	0,0000	A	tRNA	-
P663	663	G	0,0016	0,0077	A	rRNA	-
P669	669	C	0,0031	0,0000	T	rRNA	-
P679	679	A	0,0016	0,0015	C	rRNA	-
P704	704	G	0,0016	0,0000	T	rRNA	-
P705	705	T	0,0031	0,0015	C	rRNA	-
P709	709	A	0,1550	0,1267	G	rRNA	-
P721	721	C	0,0000	0,0015	T	rRNA	-

P742	742	C	0,0016	0,0000	T	rRNA	-
P745	745	G	0,0016	0,0000	A	rRNA	-
P750	750	A	0,0341	0,0186	G	rRNA	-
P751	751	G	0,0016	0,0031	A	rRNA	-
P752	752	T	0,0031	0,0000	C	rRNA	-
P769	769	A	0,0016	0,0015	G	rRNA	-
P789	789	C	0,0047	0,0000	T	rRNA	-
P794	794	C	0,0016	0,0000	T	rRNA	-
P795	795	T	0,0000	0,0015	A	rRNA	-
P813	813	G	0,0031	0,0015	A	rRNA	-
P824	824	C	0,0047	0,0015	T	rRNA	-
P825	825	A	0,0000	0,0015	T	rRNA	-
P827	827	G	0,0000	0,0015	A	rRNA	-
P896	896	G	0,0016	0,0031	A	rRNA	-
P930	930	A	0,0465	0,0247	G	rRNA	-
P934	934	C	0,0016	0,0000	G	rRNA	-
P951	951	A	0,0217	0,0201	G	rRNA	-
P955	955	C	0,0016	0,0015	A	rRNA	-
P958	958	T	0,0016	0,0000	C	rRNA	-
P980	980	C	0,0031	0,0046	T	rRNA	-
P990	990	C	0,0000	0,0031	T	rRNA	-
P1005	1005	C	0,0000	0,0015	T	rRNA	-
P1007	1007	A	0,0000	0,0015	G	rRNA	-
P1008	1008	G	0,0000	0,0015	A	rRNA	-
P1018	1018	A	0,0031	0,0015	G	rRNA	-
P1031	1031	A	0,0016	0,0000	G	rRNA	-
P1040	1040	C	0,0000	0,0015	T	rRNA	-
P1053	1053	G	0,0000	0,0015	A	rRNA	-
P1107	1107	C	0,0031	0,0000	T	rRNA	-
P1187	1187	C	0,0000	0,0015	T	rRNA	-
P1189	1189	C	0,0403	0,0665	T	rRNA	-
P1243	1243	C	0,0248	0,0170	T	rRNA	-
P1284	1284	C	0,0000	0,0015	T	rRNA	-

P1303	1303	A	0,0000	0,0031	G	rRNA	-
P1341	1341	T	0,0078	0,0046	C	rRNA	-
P1391	1391	C	0,0016	0,0015	T	rRNA	-
P1393	1393	A	0,0047	0,0046	G	rRNA	-
P1406	1406	C	0,0016	0,0031	T	rRNA	-
P1415	1415	A	0,0000	0,0015	G	rRNA	-
P1420	1420	C	0,0016	0,0000	T	rRNA	-
P1438	1438	A	0,0605	0,0386	G	rRNA	-
P1452	1452	C	0,0000	0,0015	T	rRNA	-
P1461	1461	C	0,0000	0,0015	A	rRNA	-
P1462	1462	A	0,0078	0,0046	G	rRNA	-
P1503	1503	A	0,0016	0,0015	G	rRNA	-
P1530	1530	G	0,0016	0,0000	A	rRNA	-
P1555	1555	G	0,0047	0,0000	A	rRNA	-
P1598	1598	A	0,0016	0,0062	G	rRNA	-
P1625	1625	G	0,0016	0,0000	A	tRNA	-
P1636	1636	G	0,0000	0,0015	A	tRNA	-
P1694	1694	C	0,0047	0,0077	T	rRNA	-
P1700	1700	C	0,0124	0,0124	T	rRNA	-
P1703	1703	T	0,0016	0,0031	C	rRNA	-
P1709	1709	T	0,0016	0,0000	G	rRNA	-
P1717	1717	C	0,0016	0,0000	T	rRNA	-
P1719	1719	A	0,0589	0,0417	G	rRNA	-
P1721	1721	T	0,0093	0,0124	C	rRNA	-
P1733	1733	T	0,0000	0,0031	C	rRNA	-
P1736	1736	G	0,0016	0,0077	A	rRNA	-
P1738	1738	C	0,0000	0,0015	T	rRNA	-
P1766	1766	C	0,0016	0,0000	T	rRNA	-
P1767	1767	A	0,0016	0,0000	G	rRNA	-
P1780	1780	C	0,0016	0,0000	T	rRNA	-
P1809	1809	C	0,0000	0,0015	T	rRNA	-
P1810	1810	G	0,0000	0,0015	A	rRNA	-
P1811	1811	G	0,0992	0,1283	A	rRNA	-

P1842	1842	G	0,0000	0,0031	A	rRNA	-
P1850	1850	C	0,0155	0,0201	T	rRNA	-
P1869	1869	C	0,0000	0,0015	A	rRNA	-
P1871	1871	G	0,0000	0,0015	A	rRNA	-
P1888	1888	A	0,0978	0,0757	G	rRNA	-
P1923	1923	T	0,0000	0,0015	C	rRNA	-
P1926	1926	G	0,0031	0,0015	A	rRNA	-
P1943	1943	G	0,0016	0,0000	A	rRNA	-
P2083	2083	C	0,0000	0,0015	T	rRNA	-
P2098	2098	A	0,0047	0,0093	G	rRNA	-
P2109	2109	G	0,0016	0,0000	A	rRNA	-
P2124	2124	G	0,0016	0,0000	A	rRNA	-
P2140	2140	A	0,0000	0,0031	G	rRNA	-
P2141	2141	C	0,0093	0,0046	T	rRNA	-
P2158	2158	C	0,0062	0,0155	T	rRNA	-
P2195	2195	G	0,0062	0,0031	A	rRNA	-
P2217	2217	T	0,0016	0,0046	C	rRNA	-
P2218	2218	T	0,0000	0,0015	C	rRNA	-
P2259	2259	T	0,0109	0,0139	C	rRNA	-
P2280	2280	T	0,0016	0,0000	C	rRNA	-
P2284	2284	A	0,0000	0,0015	C	rRNA	-
P2294	2294	G	0,0062	0,0046	A	rRNA	-
P2352	2352	C	0,0000	0,0046	T	rRNA	-
P2361	2361	A	0,0016	0,0015	G	rRNA	-
P2380	2380	T	0,0016	0,0000	C	rRNA	-
P2387	2387	C	0,0078	0,0046	T	rRNA	-
P2392	2392	C	0,0016	0,0000	T	rRNA	-
P2395	2395	G	0,0016	0,0000	A	rRNA	-
P2396	2396	T	0,0000	0,0031	C	rRNA	-
P2404	2404	C	0,0016	0,0000	T	rRNA	-
P2412	2412	G	0,0016	0,0000	A	rRNA	-
P2413	2413	T	0,0000	0,0015	C	rRNA	-
P2416	2416	C	0,0016	0,0000	T	rRNA	-

P2442	2442	C	0,0031	0,0031	T	rRNA	-
P2443	2443	T	0,0000	0,0015	C	rRNA	-
P2445	2445	C	0,0016	0,0000	T	rRNA	-
P2483	2483	C	0,0016	0,0015	T	rRNA	-
P2581	2581	G	0,0031	0,0077	A	rRNA	-
P2626	2626	C	0,0047	0,0000	T	rRNA	-
P2628	2628	C	0,0000	0,0015	T	rRNA	-
P2639	2639	T	0,0016	0,0031	C	rRNA	-
P2702	2702	A	0,0016	0,0000	G	rRNA	-
P2706	2706	A	0,4822	0,4250	G	rRNA	-
P2707	2707	G	0,0031	0,0031	A	rRNA	-
P2736	2736	T	0,0031	0,0000	C	rRNA	-
P2746	2746	C	0,0016	0,0000	T	rRNA	-
P2749	2749	G	0,0047	0,0015	A	rRNA	-
P2757	2757	G	0,0093	0,0031	A	rRNA	-
P2758	2758	A	0,0000	0,0015	G	rRNA	-
P2765	2765	G	0,0000	0,0015	A	rRNA	-
P2768	2768	G	0,0000	0,0015	A	rRNA	-
P2772	2772	T	0,0016	0,0000	C	rRNA	-
P2780	2780	T	0,0016	0,0015	C	rRNA	-
P2789	2789	T	0,0016	0,0000	C	rRNA	-
P2792	2792	G	0,0000	0,0015	A	rRNA	-
P2831	2831	A	0,0016	0,0046	G	rRNA	-
P2833	2833	G	0,0016	0,0000	A	rRNA	-
P2834	2834	T	0,0000	0,0015	C	rRNA	-
P2835	2835	A	0,0016	0,0000	C	rRNA	-
P2848	2848	G	0,0000	0,0015	A	rRNA	-
P2850	2850	C	0,0016	0,0046	T	rRNA	-
P2851	2851	G	0,0016	0,0000	A	rRNA	-
P2882	2882	C	0,0000	0,0015	T	rRNA	-
P2885	2885	C	0,0000	0,0015	T	rRNA	-
P2969	2969	G	0,0016	0,0000	A	rRNA	-
P3010	3010	A	0,2558	0,2581	G	rRNA	-

P3027	3027	C	0,0000	0,0015	T	rRNA	-
P3057	3057	G	0,0016	0,0000	C	rRNA	-
P3099	3099	T	0,0016	0,0000	C	rRNA	-
P3105	3105	G	0,0016	0,0000	A	rRNA	-
P3116	3116	T	0,0047	0,0046	C	rRNA	-
P3145	3145	G	0,0000	0,0031	A	rRNA	-
P3150	3150	C	0,0000	0,0015	T	rRNA	-
P3192	3192	T	0,0000	0,0031	C	rRNA	-
P3196	3196	A	0,0000	0,0015	G	rRNA	-
P3197	3197	C	0,0729	0,1020	T	rRNA	-
P3199	3199	C	0,0000	0,0015	T	rRNA	-
P3203	3203	G	0,0000	0,0015	A	rRNA	-
P3204	3204	T	0,0016	0,0000	C	rRNA	-
P3212	3212	T	0,0031	0,0031	C	rRNA	-
P3213	3213	G	0,0016	0,0000	A	rRNA	-
P3217	3217	G	0,0016	0,0000	A	rRNA	-
P3221	3221	G	0,0016	0,0000	A	rRNA	-
P3261	3261	G	0,0000	0,0015	A	tRNA	-
P3275	3275	A	0,0016	0,0000	C	tRNA	-
P3290	3290	C	0,0016	0,0000	T	tRNA	-
P3308	3308	C	0,0000	0,0046	T	ND1	non-syn:M-T
P3333	3333	T	0,0078	0,0015	C	ND1	syn:L-L
P3335	3335	C	0,0047	0,0000	T	ND1	non-syn:I-T
P3336	3336	C	0,0031	0,0000	T	ND1	syn:I-L
P3337	3337	A	0,0031	0,0000	G	ND1	non-syn:V-M
P3338	3338	C	0,0016	0,0046	T	ND1	non-syn:V-A
P3342	3342	T	0,0031	0,0015	C	ND1	syn:P-P
P3348	3348	G	0,0047	0,0015	A	ND1	syn:L-L
P3350	3350	C	0,0062	0,0000	T	ND1	non-syn:I-T
P3355	3355	G	0,0016	0,0046	A	ND1	non-syn:M-V
P3357	3357	A	0,0000	0,0015	G	ND1	syn:M-M
P3360	3360	G	0,0016	0,0015	A	ND1	syn:A-A
P3387	3387	C	0,0016	0,0000	T	ND1	syn:I-I

P3388	3388	A	0,0031	0,0000	C	ND1	non-syn:L-M
P3394	3394	C	0,0031	0,0201	T	ND1	non-syn:Y-H
P3396	3396	C	0,0016	0,0031	T	ND1	syn:Y-Y
P3398	3398	C	0,0047	0,0015	T	ND1	non-syn:M-T
P3402	3402	G	0,0000	0,0015	A	ND1	syn:Q-Q
P3421	3421	A	0,0000	0,0046	G	ND1	non-syn:V-I
P3434	3434	G	0,0031	0,0015	A	ND1	non-syn:Y-C
P3447	3447	G	0,0140	0,0093	A	ND1	syn
P3456	3456	C	0,0016	0,0000	T	ND1	syn
P3463	3463	G	0,0016	0,0000	A	ND1	non-syn:M-V
P3465	3465	G	0,0000	0,0015	A	ND1	syn
P3480	3480	G	0,0574	0,0804	A	ND1	syn
P3483	3483	A	0,0031	0,0015	G	ND1	syn
P3487	3487	A	0,0016	0,0000	C	ND1	non-syn:L-M
P3496	3496	A	0,0016	0,0015	G	ND1	non-syn:A-T
P3505	3505	G	0,0233	0,0186	A	ND1	non-syn:T-A
P3510	3510	T	0,0016	0,0000	C	ND1	syn:I-I
P3511	3511	G	0,0031	0,0000	A	ND1	non-syn:T-A
P3525	3525	A	0,0000	0,0015	C	ND1	syn
P3531	3531	A	0,0031	0,0046	G	ND1	syn
P3535	3535	C	0,0000	0,0031	T	ND1	syn:L-L
P3540	3540	C	0,0031	0,0000	T	ND1	syn:A-A
P3546	3546	T	0,0000	0,0015	C	ND1	syn
P3549	3549	T	0,0093	0,0093	C	ND1	syn
P3565	3565	G	0,0016	0,0000	A	ND1	non-syn:T-A
P3591	3591	A	0,0062	0,0031	G	ND1	syn
P3594	3594	T	0,0031	0,0031	C	ND1	syn
P3618	3618	C	0,0016	0,0000	T	ND1	syn
P3624	3624	G	0,0016	0,0000	A	ND1	syn:L-L
P3633	3633	C	0,0000	0,0031	T	ND1	syn:S-S
P3644	3644	C	0,0031	0,0015	T	ND1	non-syn:V-A
P3645	3645	C	0,0000	0,0015	T	ND1	syn
P3654	3654	T	0,0000	0,0015	C	ND1	syn

P3666	3666	A	0,0000	0,0031	G	ND1	syn:G-G
P3693	3693	A	0,0000	0,0031	G	ND1	syn
P3696	3696	T	0,0016	0,0000	C	ND1	syn
P3702	3702	G	0,0031	0,0000	A	ND1	syn
P3705	3705	A	0,0016	0,0015	G	ND1	syn
P3720	3720	G	0,0140	0,0093	A	ND1	syn
P3736	3736	A	0,0000	0,0015	G	ND1	non-syn:V-I
P3738	3738	T	0,0031	0,0031	C	ND1	syn
P3741	3741	T	0,0031	0,0031	C	ND1	syn
P3745	3745	A	0,0047	0,0000	G	ND1	non-syn:A-T
P3746	3746	T	0,0000	0,0046	C	ND1	non-syn:A-L
P3750	3750	T	0,0016	0,0000	C	ND1	syn
P3760	3760	G	0,0000	0,0046	T	ND1	non-syn:S-A
P3796	3796	G	0,0078	0,0046	A	ND1	non-syn:T-A
P3803	3803	C	0,0016	0,0000	T	ND1	non-syn:I-T
P3808	3808	G	0,0000	0,0046	A	ND1	non-syn:T-A
P3826	3826	C	0,0000	0,0062	T	ND1	syn
P3834	3834	A	0,0062	0,0031	G	ND1	syn:L-L
P3847	3847	C	0,0062	0,0062	T	ND1	syn
P3849	3849	A	0,0078	0,0062	G	ND1	syn
P3852	3852	T	0,0016	0,0000	C	ND1	syn
P3867	3867	T	0,0000	0,0015	C	ND1	syn
P3882	3882	A	0,0016	0,0015	G	ND1	syn
P3903	3903	T	0,0016	0,0000	C	ND1	syn:D-D
P3912	3912	G	0,0000	0,0015	A	ND1	syn
P3915	3915	A	0,0217	0,0371	G	ND1	syn
P3918	3918	A	0,0000	0,0031	G	ND1	syn
P3921	3921	A	0,0016	0,0031	C	ND1	syn
P3930	3930	T	0,0000	0,0015	C	ND1	syn:V-V
P3936	3936	T	0,0016	0,0000	C	ND1	syn
P3970	3970	T	0,0016	0,0031	C	ND1	syn
P3984	3984	T	0,0031	0,0000	C	ND1	syn
P3990	3990	T	0,0078	0,0015	C	ND1	syn

P3991	3991	G	0,0016	0,0046	A	ND1	non-syn:T-A
P3992	3992	T	0,0217	0,0263	C	ND1	non-syn:T-M
P4005	4005	G	0,0000	0,0015	A	ND1	syn:M-M
P4014	4014	T	0,0000	0,0015	C	ND1	syn:T-T
P4021	4021	T	0,0016	0,0000	A	ND1	non-syn:T-S
P4024	4024	G	0,0124	0,0170	A	ND1	non-syn:T-A
P4025	4025	T	0,0031	0,0000	C	ND1	non-syn:T-M
P4026	4026	G	0,0016	0,0015	A	ND1	syn
P4029	4029	A	0,0016	0,0000	C	ND1	non-syn:I-M
P4047	4047	C	0,0000	0,0015	T	ND1	syn
P4056	4056	T	0,0016	0,0000	C	ND1	syn
P4059	4059	T	0,0093	0,0062	C	ND1	syn
P4080	4080	C	0,0000	0,0031	T	ND1	syn
P4093	4093	G	0,0016	0,0000	A	ND1	non-syn:T-A
P4104	4104	G	0,0016	0,0015	A	ND1	syn:L-L
P4129	4129	G	0,0016	0,0000	A	ND1	non-syn:T-A
P4131	4131	G	0,0016	0,0000	A	ND1	syn
P4136	4136	G	0,0016	0,0000	A	ND1	non-syn:Y-C
P4143	4143	C	0,0016	0,0000	A	ND1	syn
P4155	4155	T	0,0016	0,0000	C	ND1	syn:D-D
P4173	4173	G	0,0016	0,0000	A	ND1	syn:L-L
P4179	4179	G	0,0016	0,0000	A	ND1	syn
P4188	4188	G	0,0016	0,0031	A	ND1	syn
P4216	4216	C	0,1922	0,2056	T	ND1	non-syn:Y-H
P4221	4221	T	0,0031	0,0000	C	ND1	syn
P4232	4232	C	0,0000	0,0046	T	ND1	non-syn:I-T
P4236	4236	G	0,0000	0,0015	A	ND1	syn
P4243	4243	G	0,0000	0,0046	A	ND1	non-syn:S-G
P4248	4248	C	0,0031	0,0077	T	ND1	syn
P4295	4295	G	0,0062	0,0124	A	tRNA	-
P4310	4310	G	0,0031	0,0000	A	tRNA	-
P4315	4315	G	0,0000	0,0015	A	tRNA	-
P4316	4316	G	0,0016	0,0000	A	tRNA	-

P4336	4336	C	0,0186	0,0077	T	tRNA	-
P4387	4387	T	0,0000	0,0015	C	tRNA	-
P4417	4417	G	0,0016	0,0000	A	tRNA	-
P4418	4418	C	0,0078	0,0077	T	tRNA	-
P4435	4435	G	0,0000	0,0015	A	tRNA	-
P4452	4452	C	0,0062	0,0062	T	tRNA	-
P4454	4454	C	0,0031	0,0062	T	tRNA	-
P4486	4486	C	0,0016	0,0000	A	ND2	non-syn:Q-P
P4491	4491	A	0,0062	0,0108	G	ND2	non-syn:V-I
P4508	4508	T	0,0000	0,0015	C	ND2	syn:I-I
P4511	4511	C	0,0000	0,0015	T	ND2	syn:F-F
P4529	4529	T	0,0202	0,0139	A	ND2	syn:T-T
P4541	4541	A	0,0016	0,0000	G	ND2	syn:S-S
P4553	4553	C	0,0078	0,0046	T	ND2	syn:F-F
P4561	4561	C	0,0109	0,0108	T	ND2	non-syn:V-A
P4577	4577	T	0,0000	0,0015	C	ND2	syn:N-N
P4580	4580	A	0,0512	0,0495	G	ND2	syn:M-M
P4592	4592	C	0,0000	0,0031	T	ND2	syn:I-I
P4596	4596	A	0,0000	0,0015	G	ND2	non-syn:V-I
P4610	4610	G	0,0000	0,0015	A	ND2	syn
P4639	4639	C	0,0078	0,0170	T	ND2	non-syn:I-T
P4640	4640	A	0,0016	0,0046	C	ND2	non-syn:I-M
P4646	4646	C	0,0217	0,0263	T	ND2	syn:Y-Y
P4659	4659	A	0,0016	0,0000	G	ND2	non-syn:A-T
P4688	4688	C	0,0016	0,0015	T	ND2	syn:A-A
P4703	4703	C	0,0062	0,0046	T	ND2	syn:N-N
P4709	4709	T	0,0016	0,0000	C	ND2	syn:L-L
P4727	4727	G	0,0171	0,0263	A	ND2	syn:M-M
P4732	4732	G	0,0093	0,0186	A	ND2	non-syn:N-S
P4733	4733	C	0,0000	0,0031	T	ND2	syn:N-N
P4736	4736	C	0,0078	0,0046	T	ND2	syn:T-T
P4745	4745	G	0,0093	0,0108	A	ND2	syn:Q-Q
P4760	4760	G	0,0000	0,0015	A	ND2	syn:M-M

P4763	4763	A	0,0016	0,0015	C	ND2	non-syn:I-M
P4767	4767	G	0,0000	0,0015	A	ND2	non-syn:M-V
P4769	4769	A	0,0620	0,0387	G	ND2	syn:M-M
P4781	4781	G	0,0000	0,0015	A	ND2	syn:M-M
P4790	4790	G	0,0016	0,0000	A	ND2	syn:G-G
P4793	4793	G	0,0310	0,0139	A	ND2	syn:M-M
P4796	4796	T	0,0016	0,0000	C	ND2	syn:A-A
P4811	4811	G	0,0000	0,0062	A	ND2	syn:W-W
P4820	4820	A	0,0000	0,0015	G	ND2	syn:E-E
P4823	4823	C	0,0016	0,0046	T	ND2	syn:V-V
P4824	4824	G	0,0016	0,0186	A	ND2	non-syn:T-A
P4838	4838	C	0,0000	0,0015	T	ND2	syn:P-P
P4841	4841	A	0,0000	0,0015	G	ND2	syn:L-L
P4856	4856	C	0,0016	0,0000	T	ND2	syn
P4859	4859	C	0,0062	0,0031	T	ND2	syn
P4883	4883	T	0,0031	0,0000	C	ND2	syn
P4913	4913	C	0,0000	0,0015	A	ND2	syn:S-S
P4917	4917	G	0,0992	0,0773	A	ND2	non-syn:N-D
P4928	4928	C	0,0031	0,0031	T	ND2	syn
P4936	4936	T	0,0000	0,0046	C	ND2	non-syn:T-I
P4955	4955	T	0,0000	0,0015	C	ND2	syn:I-I
P4958	4958	G	0,0000	0,0015	A	ND2	syn
P4960	4960	T	0,0016	0,0031	C	ND2	non-syn:A-V
P4961	4961	G	0,0016	0,0000	A	ND2	syn
P4991	4991	A	0,0000	0,0015	G	ND2	syn
P5003	5003	T	0,0016	0,0000	C	ND2	syn:I-I
P5004	5004	C	0,0140	0,0201	T	ND2	syn
P5036	5036	G	0,0000	0,0031	A	ND2	syn:W-W
P5046	5046	A	0,0233	0,0186	G	ND2	non-syn:V-I
P5048	5048	C	0,0000	0,0015	T	ND2	syn
P5051	5051	G	0,0016	0,0015	A	ND2	syn
P5054	5054	A	0,0031	0,0000	G	ND2	syn
P5063	5063	C	0,0000	0,0015	T	ND2	syn

P5074	5074	C	0,0016	0,0000	T	ND2	non-syn:I-T
P5082	5082	C	0,0031	0,0031	T	ND2	syn
P5095	5095	C	0,0016	0,0015	T	ND2	non-syn:I-T
P5105	5105	C	0,0016	0,0000	T	ND2	syn:T-T
P5123	5123	C	0,0000	0,0031	A	ND2	syn
P5135	5135	T	0,0016	0,0000	C	ND2	syn:N-N
P5147	5147	A	0,0496	0,0294	G	ND2	syn
P5153	5153	G	0,0000	0,0015	A	ND2	syn
P5165	5165	T	0,0016	0,0000	C	ND2	syn
P5177	5177	A	0,0000	0,0015	G	ND2	syn
P5178	5178	A	0,0031	0,0000	C	ND2	non-syn:L-M
P5187	5187	T	0,0062	0,0046	C	ND2	syn
P5194	5194	T	0,0016	0,0186	C	ND2	non-syn:P-L
P5198	5198	G	0,0078	0,0031	A	ND2	syn
P5206	5206	G	0,0016	0,0000	C	ND2	non-syn:S-C
P5231	5231	A	0,0016	0,0046	G	ND2	syn
P5237	5237	A	0,0000	0,0031	G	ND2	syn
P5240	5240	G	0,0016	0,0031	A	ND2	syn
P5250	5250	C	0,0000	0,0015	T	ND2	syn
P5261	5261	A	0,0047	0,0000	G	ND2	syn
P5262	5262	A	0,0000	0,0046	G	ND2	non-syn:A-T
P5263	5263	T	0,0062	0,0186	C	ND2	non-syn:A-V
P5267	5267	C	0,0000	0,0015	T	ND2	syn
P5277	5277	C	0,0031	0,0186	T	ND2	non-syn:F-L
P5290	5290	G	0,0000	0,0046	A	ND2	non-syn:M-S
P5291	5291	C	0,0016	0,0000	T	ND2	syn
P5301	5301	G	0,0031	0,0000	A	ND2	non-syn:I-V
P5302	5302	C	0,0000	0,0015	T	ND2	non-syn:I-T
P5307	5307	G	0,0000	0,0046	A	ND2	non-syn:T-A
P5309	5309	G	0,0016	0,0015	C	ND2	syn
P5315	5315	G	0,0031	0,0000	A	ND2	syn
P5322	5322	C	0,0031	0,0015	A	ND2	non-syn:I-L
P5345	5345	T	0,0000	0,0015	C	ND2	syn:F-F

P5348	5348	T	0,0047	0,0062	C	ND2	syn
P5351	5351	G	0,0016	0,0000	A	ND2	syn
P5360	5360	T	0,0031	0,0031	C	ND2	syn
P5375	5375	T	0,0016	0,0000	C	ND2	syn
P5378	5378	G	0,0016	0,0031	A	ND2	syn
P5390	5390	G	0,0140	0,0093	A	ND2	syn
P5393	5393	C	0,0000	0,0015	T	ND2	syn
P5423	5423	G	0,0016	0,0000	A	ND2	syn:E-E
P5426	5426	C	0,0171	0,0186	T	ND2	syn
P5427	5427	G	0,0000	0,0015	A	ND2	non-syn:T-A
P5437	5437	T	0,0016	0,0000	C	ND2	non-syn:T-I
P5440	5440	T	0,0000	0,0046	C	ND2	non-syn:P-L
P5441	5441	C	0,0016	0,0000	A	ND2	syn:P-P
P5442	5442	C	0,0016	0,0000	T	ND2	non-syn:F-L
P5460	5460	A	0,0481	0,0556	G	ND2	non-syn:A-T
P5465	5465	C	0,0031	0,0000	T	ND2	syn
P5471	5471	A	0,0047	0,0093	G	ND2	syn:T-T
P5480	5480	G	0,0016	0,0000	A	ND2	syn
P5492	5492	C	0,0016	0,0015	T	ND2	syn
P5495	5495	C	0,0155	0,0139	T	ND2	syn
P5498	5498	G	0,0031	0,0031	A	ND2	syn
P5501	5501	G	0,0016	0,0000	A	ND2	syn
P5527	5527	G	0,0016	0,0000	A	tRNA	-
P5553	5553	C	0,0016	0,0000	T	tRNA	-
P5557	5557	C	0,0031	0,0000	T	tRNA	-
P5558	5558	G	0,0016	0,0000	A	tRNA	-
P5563	5563	A	0,0031	0,0000	G	tRNA	-
P5567	5567	C	0,0000	0,0015	T	tRNA	-
P5580	5580	C	0,0047	0,0000	T	Non-Coding	-
P5581	5581	G	0,0062	0,0000	A	Non-Coding	-
P5582	5582	G	0,0000	0,0015	A	Non-Coding	-
P5585	5585	A	0,0047	0,0015	G	Non-Coding	-
P5586	5586	T	0,0016	0,0015	C	Non-Coding	-

P5592	5592	G	0,0016	0,0000	A	tRNA	-
P5600	5600	G	0,0000	0,0015	A	tRNA	-
P5618	5618	C	0,0078	0,0000	T	tRNA	-
P5628	5628	C	0,0000	0,0015	T	tRNA	-
P5633	5633	T	0,0062	0,0062	C	tRNA	-
P5639	5639	G	0,0000	0,0015	A	tRNA	-
P5655	5655	C	0,0000	0,0015	T	tRNA	-
P5656	5656	G	0,0264	0,0201	A	Non-Coding	-
P5746	5746	A	0,0016	0,0046	G	tRNA	-
P5755	5755	G	0,0016	0,0000	C	tRNA	-
P5772	5772	A	0,0031	0,0000	G	tRNA	-
P5773	5773	A	0,0047	0,0046	G	tRNA	-
P5785	5785	C	0,0016	0,0031	T	tRNA	-
P5806	5806	C	0,0000	0,0015	T	tRNA	-
P5811	5811	G	0,0016	0,0031	A	tRNA	-
P5812	5812	G	0,0000	0,0015	A	tRNA	-
P5821	5821	A	0,0031	0,0000	G	tRNA	-
P5823	5823	G	0,0016	0,0015	A	tRNA	-
P5824	5824	A	0,0016	0,0046	G	tRNA	-
P5839	5839	T	0,0000	0,0031	C	tRNA	-
P5843	5843	G	0,0000	0,0015	A	tRNA	-
P5894	5894	G	0,0016	0,0000	A	Non-Coding	-
P5913	5913	A	0,0078	0,0093	G	CO1	non-syn:D-N
P5915	5915	T	0,0000	0,0015	C	CO1	syn
P5933	5933	G	0,0016	0,0000	A	CO1	syn:T-T
P5978	5978	G	0,0062	0,0062	A	CO1	syn
P5984	5984	G	0,0016	0,0000	A	CO1	syn
P5999	5999	C	0,0171	0,0232	T	CO1	syn
P6002	6002	G	0,0016	0,0000	A	CO1	syn
P6009	6009	T	0,0000	0,0015	C	CO1	non-syn:L-F
P6014	6014	C	0,0016	0,0000	T	CO1	syn
P6023	6023	A	0,0016	0,0015	G	CO1	syn
P6026	6026	A	0,0000	0,0015	G	CO1	syn

P6038	6038	T	0,0000	0,0015	C	CO1	syn:G-G
P6040	6040	G	0,0031	0,0000	A	CO1	non-syn:N-S
P6045	6045	T	0,0140	0,0093	C	CO1	syn
P6047	6047	G	0,0171	0,0232	A	CO1	syn
P6050	6050	C	0,0000	0,0046	T	CO1	syn
P6054	6054	T	0,0016	0,0000	G	CO1	non-syn:D-Y
P6071	6071	C	0,0000	0,0015	T	CO1	syn
P6146	6146	G	0,0000	0,0062	A	CO1	syn
P6152	6152	C	0,0140	0,0108	T	CO1	syn
P6164	6164	T	0,0016	0,0015	C	CO1	syn
P6179	6179	A	0,0016	0,0000	G	CO1	syn
P6182	6182	A	0,0000	0,0015	G	CO1	syn
P6185	6185	C	0,0000	0,0015	T	CO1	syn:F-F
P6221	6221	C	0,0202	0,0232	T	CO1	syn
P6231	6231	T	0,0016	0,0000	C	CO1	syn
P6248	6248	C	0,0000	0,0015	T	CO1	syn
P6249	6249	A	0,0016	0,0000	G	CO1	non-syn:A-T
P6253	6253	C	0,0078	0,0015	T	CO1	non-syn:M-T
P6254	6254	G	0,0031	0,0031	A	CO1	syn
P6260	6260	A	0,0078	0,0124	G	CO1	syn:E-E
P6261	6261	A	0,0062	0,0046	G	CO1	non-syn:A-T
P6267	6267	A	0,0000	0,0015	G	CO1	non-syn:A-T
P6272	6272	G	0,0031	0,0000	A	CO1	syn
P6284	6284	G	0,0016	0,0000	A	CO1	syn
P6287	6287	A	0,0000	0,0015	C	CO1	syn:V-V
P6293	6293	C	0,0000	0,0062	T	CO1	syn
P6299	6299	G	0,0047	0,0000	A	CO1	syn:L-L
P6340	6340	T	0,0016	0,0000	C	CO1	non-syn:T-I
P6345	6345	C	0,0016	0,0000	T	CO1	non-syn:F-L
P6365	6365	C	0,0109	0,0124	T	CO1	syn
P6366	6366	A	0,0016	0,0031	G	CO1	non-syn:V-I
P6371	6371	T	0,0202	0,0216	C	CO1	syn
P6374	6374	C	0,0000	0,0015	T	CO1	syn

P6378	6378	C	0,0016	0,0000	T	CO1	syn
P6383	6383	A	0,0031	0,0046	G	CO1	syn
P6392	6392	C	0,0016	0,0046	T	CO1	syn
P6398	6398	T	0,0031	0,0000	C	CO1	syn:I-I
P6407	6407	C	0,0031	0,0000	T	CO1	syn
P6410	6410	T	0,0031	0,0000	C	CO1	syn
P6423	6423	G	0,0000	0,0015	C	CO1	non-syn:P-A
P6426	6426	A	0,0000	0,0015	G	CO1	non-syn:A-T
P6428	6428	T	0,0016	0,0000	C	CO1	syn:A-A
P6431	6431	G	0,0000	0,0015	A	CO1	syn:M-M
P6436	6436	C	0,0000	0,0015	A	CO1	non-syn:Q-P
P6445	6445	T	0,0016	0,0031	C	CO1	non-syn:T-M
P6446	6446	A	0,0031	0,0031	G	CO1	syn
P6448	6448	A	0,0000	0,0015	C	CO1	non-syn:P-H
P6455	6455	T	0,0031	0,0031	C	CO1	syn
P6464	6464	A	0,0016	0,0015	C	CO1	syn
P6465	6465	A	0,0062	0,0000	G	CO1	non-syn:V-I
P6473	6473	T	0,0016	0,0000	C	CO1	syn
P6480	6480	A	0,0016	0,0046	G	CO1	non-syn:V-I
P6489	6489	A	0,0031	0,0077	C	CO1	non-syn:L-I
P6506	6506	T	0,0016	0,0000	C	CO1	syn:V-V
P6515	6515	G	0,0031	0,0015	T	CO1	syn:A-A
P6518	6518	T	0,0078	0,0046	C	CO1	syn
P6524	6524	C	0,0016	0,0015	T	CO1	syn
P6527	6527	G	0,0000	0,0093	A	CO1	syn
P6528	6528	T	0,0016	0,0015	C	CO1	syn
P6531	6531	T	0,0016	0,0000	C	CO1	syn
P6539	6539	T	0,0016	0,0000	C	CO1	syn
P6546	6546	T	0,0047	0,0015	C	CO1	non-syn:L-F
P6548	6548	T	0,0000	0,0015	C	CO1	syn
P6554	6554	T	0,0016	0,0015	C	CO1	syn
P6557	6557	T	0,0016	0,0015	C	CO1	syn
P6599	6599	G	0,0047	0,0015	A	CO1	syn

P6614	6614	C	0,0000	0,0015	T	CO1	syn
P6617	6617	T	0,0016	0,0000	C	CO1	syn
P6620	6620	C	0,0000	0,0015	T	CO1	syn
P6629	6629	G	0,0000	0,0031	A	CO1	syn
P6635	6635	C	0,0016	0,0031	T	CO1	syn
P6650	6650	G	0,0000	0,0015	A	CO1	syn:P-P
P6665	6665	T	0,0000	0,0015	C	CO1	syn
P6671	6671	C	0,0078	0,0062	T	CO1	syn
P6680	6680	C	0,0047	0,0031	T	CO1	syn:T-T
P6713	6713	T	0,0000	0,0015	C	CO1	syn
P6719	6719	C	0,0016	0,0031	T	CO1	syn
P6722	6722	A	0,0000	0,0015	G	CO1	syn
P6731	6731	C	0,0016	0,0000	T	CO1	syn:A-A
P6734	6734	A	0,0109	0,0062	G	CO1	syn
P6750	6750	T	0,0016	0,0000	C	CO1	syn
P6776	6776	C	0,0248	0,0371	T	CO1	syn
P6806	6806	G	0,0000	0,0015	A	CO1	syn
P6812	6812	G	0,0016	0,0000	A	CO1	syn
P6827	6827	C	0,0000	0,0015	T	CO1	syn:A-A
P6845	6845	T	0,0000	0,0015	C	CO1	syn
P6851	6851	T	0,0016	0,0000	C	CO1	syn:T-T
P6869	6869	T	0,0000	0,0015	C	CO1	syn
P6890	6890	G	0,0016	0,0000	A	CO1	syn:G-G
P6899	6899	A	0,0016	0,0015	G	CO1	syn
P6915	6915	A	0,0000	0,0031	G	CO1	non-syn:V-M
P6927	6927	T	0,0000	0,0015	C	CO1	syn:L-L
P6929	6929	G	0,0016	0,0015	A	CO1	syn
P6938	6938	T	0,0016	0,0000	C	CO1	syn
P6962	6962	T	0,0016	0,0000	G	CO1	syn
P6989	6989	G	0,0000	0,0015	A	CO1	syn
P7022	7022	C	0,0016	0,0046	T	CO1	syn
P7025	7025	G	0,0016	0,0000	A	CO1	syn
P7028	7028	C	0,4884	0,4297	T	CO1	syn:A-A

P7041	7041	A	0,0016	0,0000	G	CO1	non-syn:V-I
P7055	7055	G	0,0016	0,0062	A	CO1	syn:G-G
P7076	7076	G	0,0000	0,0015	A	CO1	syn:G-G
P7080	7080	C	0,0000	0,0015	T	CO1	non-syn:F-L
P7100	7100	G	0,0016	0,0000	A	CO1	syn:L-L
P7118	7118	G	0,0000	0,0015	A	CO1	syn
P7119	7119	A	0,0016	0,0015	G	CO1	non-syn:D-N
P7146	7146	G	0,0000	0,0015	A	CO1	non-syn:T-A
P7148	7148	C	0,0000	0,0015	T	CO1	syn
P7149	7149	G	0,0016	0,0000	A	CO1	non-syn:I-V
P7169	7169	C	0,0000	0,0015	T	CO1	syn:N-N
P7175	7175	C	0,0016	0,0000	T	CO1	syn
P7184	7184	G	0,0016	0,0031	A	CO1	syn
P7193	7193	C	0,0016	0,0000	T	CO1	syn
P7202	7202	G	0,0016	0,0000	A	CO1	syn
P7211	7211	A	0,0016	0,0000	G	CO1	syn
P7220	7220	C	0,0016	0,0031	T	CO1	syn
P7226	7226	A	0,0000	0,0062	G	CO1	syn
P7245	7245	G	0,0000	0,0015	A	CO1	non-syn:T-A
P7247	7247	T	0,0031	0,0000	C	CO1	syn
P7256	7256	T	0,0016	0,0015	C	CO1	syn
P7262	7262	G	0,0016	0,0000	A	CO1	syn
P7268	7268	C	0,0000	0,0015	T	CO1	syn:S-S
P7269	7269	A	0,0016	0,0015	G	CO1	non-syn:V-M
P7270	7270	C	0,0016	0,0046	T	CO1	non-syn:V-A
P7274	7274	T	0,0016	0,0015	C	CO1	syn
P7280	7280	T	0,0000	0,0015	C	CO1	syn:F-F
P7289	7289	G	0,0016	0,0000	A	CO1	syn:L-L
P7299	7299	G	0,0016	0,0031	A	CO1	non-syn:M-V
P7302	7302	C	0,0016	0,0000	T	CO1	syn
P7309	7309	C	0,0062	0,0062	T	CO1	non-syn:I-T
P7337	7337	A	0,0062	0,0062	G	CO1	syn
P7340	7340	A	0,0016	0,0015	G	CO1	syn

P7372	7372	C	0,0000	0,0015	T	CO1	non-syn:M-T
P7385	7385	G	0,0171	0,0108	A	CO1	syn
P7389	7389	C	0,0016	0,0015	T	CO1	non-syn:Y-H
P7403	7403	G	0,0000	0,0015	A	CO1	syn
P7424	7424	G	0,0016	0,0015	A	CO1	syn
P7430	7430	G	0,0000	0,0015	A	CO1	syn
P7444	7444	A	0,0093	0,0062	G	CO1	non-syn:Term-K
P7476	7476	T	0,0217	0,0294	C	tRNA	-
P7501	7501	C	0,0031	0,0031	T	tRNA	-
P7517	7517	G	0,0000	0,0015	A	Non-Coding	-
P7521	7521	A	0,0031	0,0046	G	tRNA	-
P7533	7533	T	0,0000	0,0015	C	tRNA	-
P7547	7547	C	0,0016	0,0015	T	tRNA	-
P7570	7570	G	0,0000	0,0015	A	tRNA	-
P7581	7581	C	0,0000	0,0015	T	tRNA	-
P7598	7598	A	0,0000	0,0046	G	CO2	non-syn:A-T
P7604	7604	A	0,0000	0,0046	G	CO2	non-syn:V-M
P7621	7621	C	0,0016	0,0000	T	CO2	syn
P7630	7630	C	0,0000	0,0015	T	CO2	syn:P-P
P7642	7642	A	0,0031	0,0000	G	CO2	syn
P7645	7645	C	0,0000	0,0031	T	CO2	syn
P7650	7650	T	0,0016	0,0000	C	CO2	non-syn:T-I
P7657	7657	C	0,0000	0,0062	T	CO2	syn
P7680	7680	C	0,0016	0,0000	T	CO2	non-syn:F-S
P7684	7684	C	0,0016	0,0000	T	CO2	syn
P7685	7685	G	0,0016	0,0000	A	CO2	non-syn:I-V
P7690	7690	T	0,0000	0,0015	C	CO2	syn:C-C
P7705	7705	C	0,0047	0,0093	T	CO2	syn
P7711	7711	C	0,0000	0,0015	T	CO2	syn
P7747	7747	T	0,0000	0,0015	C	CO2	syn
P7754	7754	A	0,0000	0,0015	G	CO2	non-syn:D-N
P7762	7762	A	0,0000	0,0046	G	CO2	syn
P7768	7768	G	0,0357	0,0386	A	CO2	syn

P7771	7771	G	0,0016	0,0000	A	CO2	syn
P7775	7775	A	0,0000	0,0031	G	CO2	non-syn:V-I
P7789	7789	A	0,0155	0,0201	G	CO2	syn
P7792	7792	T	0,0000	0,0015	C	CO2	syn
P7804	7804	G	0,0016	0,0000	A	CO2	syn
P7805	7805	A	0,0062	0,0046	G	CO2	non-syn:V-I
P7807	7807	A	0,0000	0,0015	C	CO2	syn:V-V
P7810	7810	T	0,0000	0,0015	C	CO2	syn
P7843	7843	G	0,0016	0,0015	A	CO2	syn
P7853	7853	A	0,0062	0,0031	G	CO2	non-syn:V-I
P7861	7861	C	0,0000	0,0015	T	CO2	syn
P7864	7864	T	0,0186	0,0124	C	CO2	syn
P7867	7867	T	0,0000	0,0015	C	CO2	syn
P7870	7870	C	0,0016	0,0000	T	CO2	syn
P7873	7873	T	0,0062	0,0046	C	CO2	syn
P7891	7891	T	0,0031	0,0015	C	CO2	syn
P7897	7897	A	0,0000	0,0015	G	CO2	syn
P7930	7930	T	0,0047	0,0077	A	CO2	syn
P7941	7941	G	0,0000	0,0046	A	CO2	non-syn:N-S
P7958	7958	T	0,0016	0,0000	C	CO2	non-syn:P-S
P7960	7960	G	0,0016	0,0000	A	CO2	syn
P7961	7961	C	0,0000	0,0062	T	CO2	syn
P7963	7963	G	0,0000	0,0015	A	CO2	syn
P7971	7971	G	0,0016	0,0000	A	CO2	non-syn:E-G
P7978	7978	T	0,0031	0,0000	C	CO2	syn
P7979	7979	A	0,0016	0,0015	G	CO2	non-syn:D-N
P7984	7984	A	0,0016	0,0015	G	CO2	syn
P8008	8008	G	0,0016	0,0000	A	CO2	syn
P8014	8014	T	0,0000	0,0015	A	CO2	syn
P8020	8020	A	0,0016	0,0000	G	CO2	syn
P8022	8022	C	0,0000	0,0015	T	CO2	non-syn:I-T
P8041	8041	G	0,0016	0,0015	A	CO2	syn:M-M
P8058	8058	G	0,0016	0,0000	A	CO2	non-syn:D-G

P8065	8065	A	0,0000	0,0015	G	CO2	syn
P8104	8104	C	0,0016	0,0000	T	CO2	syn
P8107	8107	T	0,0016	0,0000	A	CO2	syn
P8108	8108	G	0,0016	0,0000	A	CO2	non-syn:I-V
P8125	8125	T	0,0016	0,0000	C	CO2	syn:N-N
P8137	8137	T	0,0031	0,0031	C	CO2	syn
P8152	8152	A	0,0016	0,0015	G	CO2	syn
P8155	8155	A	0,0000	0,0015	G	CO2	syn
P8164	8164	T	0,0016	0,0000	C	CO2	syn
P8167	8167	C	0,0000	0,0031	T	CO2	syn
P8179	8179	G	0,0031	0,0000	A	CO2	syn
P8185	8185	C	0,0016	0,0000	T	CO2	syn:C-C
P8200	8200	C	0,0016	0,0000	T	CO2	syn
P8206	8206	A	0,0016	0,0000	G	CO2	syn
P8212	8212	T	0,0016	0,0000	C	CO2	syn:I-I
P8216	8216	T	0,0000	0,0015	C	CO2	syn:L-L
P8222	8222	C	0,0000	0,0031	T	CO2	syn
P8230	8230	T	0,0000	0,0031	C	CO2	syn
P8242	8242	C	0,0016	0,0000	T	CO2	syn
P8245	8245	G	0,0047	0,0015	A	CO2	syn
P8248	8248	G	0,0000	0,0015	A	CO2	syn
P8251	8251	A	0,0465	0,0340	G	CO2	syn
P8256	8256	C	0,0031	0,0000	T	CO2	non-syn:V-A
P8260	8260	C	0,0031	0,0000	T	CO2	syn
P8261	8261	G	0,0000	0,0015	A	CO2	non-syn:T-A
P8269	8269	A	0,0202	0,0340	G	CO2	non-coding
P8271	8271	T	0,0094	0,0031	A	Non-Coding	-
P8276	8276	G	0,0000	0,0016	C	Non-Coding	-
P8286	8286	C	0,0031	0,0031	T	Non-Coding	-
P8292	8292	A	0,0016	0,0015	G	Non-Coding	-
P8302	8302	G	0,0016	0,0000	A	tRNA	-
P8308	8308	G	0,0078	0,0046	A	tRNA	-
P8383	8383	C	0,0016	0,0000	T	ATP8	syn

P8388	8388	C	0,0016	0,0015	T	ATP8	non-syn:V-A
P8393	8393	T	0,0078	0,0046	C	ATP8	non-syn:P-S
P8395	8395	T	0,0000	0,0015	C	ATP8	syn
P8410	8410	T	0,0000	0,0015	C	ATP8	syn
P8416	8416	T	0,0000	0,0015	C	ATP8	syn
P8425	8425	G	0,0031	0,0015	A	ATP8	syn:L-L
P8426	8426	C	0,0000	0,0031	T	ATP8	non-syn:F-L
P8432	8432	C	0,0016	0,0000	A	ATP8	non-syn:I-L
P8448	8448	C	0,0124	0,0108	T	ATP8	non-syn:M-T
P8462	8462	C	0,0016	0,0000	T	ATP8	non-syn:Y-H
P8468	8468	T	0,0000	0,0015	C	ATP8	syn
P8470	8470	G	0,0031	0,0015	A	ATP8	syn
P8472	8472	T	0,0016	0,0031	C	ATP8	non-syn:P-L
P8473	8473	C	0,0140	0,0263	T	ATP8	syn
P8485	8485	A	0,0000	0,0015	G	ATP8	syn
P8492	8492	G	0,0016	0,0015	A	ATP8	non-syn:K-E
P8496	8496	C	0,0000	0,0015	T	ATP8	non-syn:M-T
P8503	8503	C	0,0016	0,0000	T	ATP8	syn
P8504	8504	C	0,0000	0,0015	T	ATP8	non-syn:Y-H
P8512	8512	G	0,0093	0,0031	A	ATP8	syn
P8519	8519	A	0,0016	0,0031	G	ATP8	non-syn:E-K
P8537	8537	G	0,0031	0,0015	A	ATP6	ATP8:I-V/ATP6:N-S
P8545	8545	A	0,0016	0,0000	G	ATP6	ATP8:S-S/ATP6:A-T
P8557	8557	A	0,0047	0,0155	G	ATP6	ATP8:L-L/ATP6:A-T
P8558	8558	T	0,0047	0,0046	C	ATP6	ATP8:L-L/ATP6:A-T
P8567	8567	C	0,0031	0,0015	T	ATP6	ATP8:P-S/ATP6:A-V
P8572	8572	A	0,0031	0,0031	G	ATP6	ATP8:P-S/ATP6:A-V
P8573	8573	A	0,0031	0,0031	G	ATP6	ATP8:S-P/ATP6:I-T
P8578	8578	T	0,0000	0,0015	C	ATP6	ATP8:S-P/ATP6:I-T
P8584	8584	A	0,0000	0,0015	G	ATP6	¯P8:Term-Term/ATP6:G-S
P8589	8589	G	0,0016	0,0000	A	ATP6	¯P8:Term-Term/ATP6:G-S
P8592	8592	A	0,0016	0,0015	G	ATP6	non-syn:G-D
P8602	8602	C	0,0078	0,0015	T	ATP6	non-syn:P-S

P8604	8604	A	0,0016	0,0000	T	ATP6	non-syn:A-T
P8610	8610	C	0,0000	0,0015	T	ATP6	syn
P8614	8614	C	0,0016	0,0000	T	ATP6	syn
P8616	8616	T	0,0078	0,0015	G	ATP6	non-syn:F-L
P8618	8618	C	0,0031	0,0000	T	ATP6	non-syn:F-L
P8631	8631	G	0,0016	0,0000	A	ATP6	syn
P8634	8634	C	0,0016	0,0046	T	ATP6	syn
P8642	8642	G	0,0000	0,0015	A	ATP6	non-syn:L-F
P8645	8645	G	0,0016	0,0000	A	ATP6	non-syn:I-T
P8648	8648	C	0,0016	0,0000	G	ATP6	syn
P8651	8651	C	0,0016	0,0000	T	ATP6	syn
P8653	8653	G	0,0000	0,0015	A	ATP6	non-syn:N-S
P8655	8655	T	0,0000	0,0015	C	ATP6	non-syn:N-S
P8659	8659	G	0,0031	0,0000	A	ATP6	non-syn:R-P
P8673	8673	G	0,0000	0,0046	A	ATP6	non-syn:L-P
P8684	8684	T	0,0031	0,0031	C	ATP6	non-syn:I-V
P8696	8696	C	0,0016	0,0000	T	ATP6	syn
P8697	8697	A	0,0947	0,0773	G	ATP6	non-syn:T-A
P8701	8701	G	0,0093	0,0077	A	ATP6	syn
P8705	8705	C	0,0062	0,0263	T	ATP6	non-syn:T-I
P8706	8706	G	0,0031	0,0015	A	ATP6	non-syn:M-T
P8711	8711	G	0,0016	0,0000	A	ATP6	syn
P8715	8715	C	0,0031	0,0062	T	ATP6	non-syn:T-A
P8718	8718	G	0,0016	0,0000	A	ATP6	non-syn:M-T
P8723	8723	A	0,0093	0,0000	G	ATP6	syn
P8736	8736	C	0,0016	0,0000	T	ATP6	non-syn:N-S
P8752	8752	G	0,0000	0,0015	A	ATP6	syn
P8762	8762	C	0,0016	0,0000	T	ATP6	syn
P8763	8763	C	0,0000	0,0015	T	ATP6	non-syn:R-Q
P8764	8764	A	0,0016	0,0000	G	ATP6	syn
P8765	8765	T	0,0031	0,0015	C	ATP6	non-syn:I-V
P8787	8787	A	0,0016	0,0000	C	ATP6	non-syn:I-T
P8790	8790	A	0,0000	0,0062	G	ATP6	syn

P8793	8793	C	0,0016	0,0000	T	ATP6	non-syn:A-T
P8794	8794	T	0,0016	0,0077	C	ATP6	non-syn:A-V
P8812	8812	G	0,0078	0,0000	A	ATP6	syn
P8814	8814	T	0,0016	0,0000	C	ATP6	syn
P8818	8818	T	0,0124	0,0077	C	ATP6	syn
P8836	8836	G	0,0016	0,0015	A	ATP6	non-syn:H-Y
P8838	8838	A	0,0031	0,0000	G	ATP6	non-syn:T-A
P8839	8839	A	0,0000	0,0015	G	ATP6	syn
P8842	8842	G	0,0000	0,0031	A	ATP6	syn
P8843	8843	C	0,0047	0,0000	T	ATP6	non-syn:M-V
P8856	8856	A	0,0016	0,0000	G	ATP6	syn
P8857	8857	A	0,0000	0,0031	G	ATP6	non-syn:A-T
P8860	8860	A	0,0295	0,0124	G	ATP6	non-syn:I-V
P8865	8865	A	0,0062	0,0031	G	ATP6	non-syn:I-T
P8868	8868	C	0,0000	0,0015	T	ATP6	syn
P8869	8869	G	0,0078	0,0170	A	ATP6	non-syn:G-S
P8870	8870	C	0,0000	0,0015	T	ATP6	non-syn:T-A
P8886	8886	A	0,0000	0,0046	G	ATP6	syn
P8887	8887	G	0,0047	0,0031	A	ATP6	syn
P8895	8895	A	0,0016	0,0015	T	ATP6	non-syn:M-V
P8898	8898	T	0,0016	0,0000	C	ATP6	non-syn:M-T
P8901	8901	G	0,0047	0,0000	A	ATP6	syn
P8904	8904	T	0,0000	0,0015	C	ATP6	non-syn:I-V
P8908	8908	C	0,0000	0,0015	T	ATP6	non-syn:N-K
P8910	8910	T	0,0016	0,0000	C	ATP6	syn
P8923	8923	G	0,0062	0,0015	A	ATP6	syn
P8928	8928	C	0,0016	0,0000	T	ATP6	syn
P8937	8937	C	0,0000	0,0031	T	ATP6	non-syn:F-L
P8939	8939	C	0,0016	0,0000	T	ATP6	syn
P8943	8943	A	0,0031	0,0000	C	ATP6	non-syn:T-A
P8944	8944	G	0,0016	0,0015	A	ATP6	syn
P8950	8950	A	0,0016	0,0046	G	ATP6	syn
P8967	8967	T	0,0000	0,0015	C	ATP6	non-syn:I-T

P8974	8974	T	0,0016	0,0000	C	ATP6	syn
P8994	8994	A	0,0264	0,0201	G	ATP6	non-syn:M-V
P9004	9004	T	0,0000	0,0015	C	ATP6	non-syn:V-I
P9006	9006	G	0,0016	0,0046	A	ATP6	syn
P9007	9007	G	0,0016	0,0000	A	ATP6	non-syn:L-F
P9031	9031	T	0,0000	0,0031	C	ATP6	syn
P9033	9033	G	0,0016	0,0015	A	ATP6	syn
P9038	9038	C	0,0000	0,0015	T	ATP6	syn
P9039	9039	A	0,0031	0,0015	G	ATP6	non-syn:T-A
P9041	9041	G	0,0000	0,0015	A	ATP6	syn:L-L
P9052	9052	G	0,0000	0,0015	A	ATP6	syn
P9053	9053	A	0,0016	0,0062	G	ATP6	non-syn:M-T
P9055	9055	A	0,0589	0,0819	G	ATP6	syn
P9058	9058	G	0,0000	0,0015	A	ATP6	non-syn:H-R
P9066	9066	G	0,0062	0,0062	A	ATP6	non-syn:S-G
P9070	9070	G	0,0000	0,0062	T	ATP6	non-syn:S-N
P9073	9073	G	0,0000	0,0015	A	ATP6	non-syn:A-T
P9088	9088	C	0,0000	0,0015	T	ATP6	non-syn:T-A
P9093	9093	G	0,0062	0,0155	A	ATP6	syn
P9096	9096	C	0,0016	0,0000	T	ATP6	non-syn:S-A
P9101	9101	C	0,0016	0,0000	T	ATP6	non-syn:T-A
P9116	9116	C	0,0016	0,0000	T	ATP6	non-syn:S-P
P9117	9117	C	0,0093	0,0046	T	ATP6	syn
P9123	9123	A	0,0124	0,0201	G	ATP6	syn
P9129	9129	T	0,0031	0,0000	C	ATP6	non-syn:I-T
P9145	9145	A	0,0047	0,0015	G	ATP6	non-syn:I-T
P9148	9148	C	0,0016	0,0000	T	ATP6	syn
P9150	9150	G	0,0124	0,0201	A	ATP6	syn
P9151	9151	G	0,0000	0,0015	A	ATP6	syn
P9165	9165	C	0,0016	0,0000	T	ATP6	non-syn:A-T
P9180	9180	G	0,0031	0,0000	A	ATP6	syn
P9181	9181	G	0,0000	0,0031	A	ATP6	syn
P9192	9192	A	0,0016	0,0015	G	ATP6	non-syn:I-V

P9196	9196	A	0,0000	0,0046	G	ATP6	syn
P9210	9210	G	0,0047	0,0000	A	ATP6	syn
P9214	9214	G	0,0016	0,0015	A	ATP6	non-syn:S-G
P9221	9221	G	0,0016	0,0000	A	ATP6	syn
P9233	9233	C	0,0031	0,0015	T	ATP6	non-syn:D-N
P9254	9254	G	0,0171	0,0077	A	CO3	non-syn:T-A
P9266	9266	A	0,0062	0,0046	G	CO3	non-syn:H-R
P9299	9299	G	0,0031	0,0000	A	CO3	syn
P9300	9300	A	0,0031	0,0031	G	CO3	syn
P9305	9305	A	0,0000	0,0015	G	CO3	syn
P9325	9325	C	0,0016	0,0000	T	CO3	syn
P9329	9329	A	0,0031	0,0000	G	CO3	syn
P9335	9335	T	0,0016	0,0031	C	CO3	non-syn:A-T
P9336	9336	G	0,0031	0,0000	A	CO3	syn
P9338	9338	G	0,0016	0,0000	A	CO3	non-syn:M-T
P9344	9344	T	0,0000	0,0015	C	CO3	syn
P9365	9365	T	0,0016	0,0031	C	CO3	syn
P9368	9368	G	0,0031	0,0000	A	CO3	non-syn:M-V
P9371	9371	T	0,0000	0,0015	C	CO3	syn
P9377	9377	G	0,0031	0,0046	A	CO3	syn
P9380	9380	A	0,0186	0,0294	G	CO3	syn
P9389	9389	G	0,0016	0,0000	A	CO3	syn
P9391	9391	T	0,0031	0,0031	C	CO3	syn
P9392	9392	G	0,0000	0,0015	A	CO3	syn
P9413	9413	T	0,0000	0,0015	C	CO3	syn
P9438	9438	A	0,0016	0,0000	G	CO3	syn
P9469	9469	T	0,0016	0,0000	C	CO3	non-syn:T-M
P9477	9477	A	0,0729	0,1005	G	CO3	syn
P9478	9478	C	0,0016	0,0000	T	CO3	syn:G-G
P9479	9479	C	0,0000	0,0015	T	CO3	non-syn:G-S
P9480	9480	C	0,0016	0,0015	T	CO3	non-syn:T-I
P9489	9489	A	0,0000	0,0015	G	CO3	non-syn:V-I
P9500	9500	G	0,0016	0,0000	C	CO3	non-syn:V-A

P9531	9531	G	0,0000	0,0015	A	CO3	syn:V-V
P9540	9540	C	0,0078	0,0062	T	CO3	non-syn:F-L
P9545	9545	G	0,0078	0,0077	A	CO3	non-syn:A-T
P9548	9548	A	0,0016	0,0077	G	CO3	non-syn:F-L
P9554	9554	A	0,0016	0,0000	G	CO3	non-syn:T-A
P9563	9563	G	0,0016	0,0000	A	CO3	syn
P9575	9575	A	0,0000	0,0031	G	CO3	syn
P9581	9581	C	0,0016	0,0015	T	CO3	syn
P9591	9591	A	0,0016	0,0015	G	CO3	syn
P9592	9592	C	0,0000	0,0015	T	CO3	syn
P9596	9596	G	0,0031	0,0000	A	CO3	syn
P9599	9599	T	0,0016	0,0000	C	CO3	syn
P9612	9612	A	0,0031	0,0046	G	CO3	non-syn:V-I
P9620	9620	T	0,0016	0,0000	C	CO3	non-syn:V-A
P9629	9629	C	0,0016	0,0000	A	CO3	syn
P9632	9632	G	0,0016	0,0046	A	CO3	syn
P9635	9635	C	0,0000	0,0015	A	CO3	non-syn:V-M
P9644	9644	G	0,0016	0,0015	A	CO3	syn
P9653	9653	C	0,0016	0,0000	T	CO3	syn
P9656	9656	C	0,0016	0,0031	T	CO3	syn
P9664	9664	G	0,0047	0,0031	A	CO3	syn
P9667	9667	G	0,0140	0,0139	A	CO3	syn
P9670	9670	G	0,0000	0,0031	A	CO3	syn
P9682	9682	C	0,0000	0,0015	T	CO3	syn
P9689	9689	G	0,0000	0,0015	A	CO3	non-syn:E-G
P9698	9698	C	0,0636	0,0819	T	CO3	non-syn:N-S
P9719	9719	A	0,0016	0,0000	C	CO3	non-syn:N-S
P9728	9728	T	0,0000	0,0015	C	CO3	non-syn:M-T
P9738	9738	A	0,0000	0,0062	G	CO3	syn
P9740	9740	T	0,0000	0,0015	C	CO3	syn
P9756	9756	G	0,0016	0,0046	T	CO3	syn
P9758	9758	C	0,0047	0,0062	T	CO3	syn:T-T
P9770	9770	C	0,0016	0,0031	T	CO3	non-syn:A-T

P9777	9777	A	0,0016	0,0000	G	CO3	syn:A-A
P9782	9782	T	0,0000	0,0031	C	CO3	non-syn:S-A
P9788	9788	G	0,0000	0,0015	C	CO3	syn
P9804	9804	A	0,0047	0,0031	G	CO3	syn
P9809	9809	G	0,0016	0,0000	A	CO3	non-syn:G-S
P9812	9812	T	0,0031	0,0015	C	CO3	syn
P9815	9815	T	0,0000	0,0015	C	CO3	syn
P9824	9824	C	0,0000	0,0031	T	CO3	non-syn:A-T
P9836	9836	C	0,0016	0,0000	T	CO3	syn:T-T
P9840	9840	A	0,0016	0,0000	T	CO3	syn
P9843	9843	G	0,0000	0,0015	A	CO3	syn
P9845	9845	C	0,0031	0,0015	T	CO3	syn
P9854	9854	C	0,0047	0,0000	T	CO3	syn
P9861	9861	C	0,0000	0,0015	T	CO3	non-syn:S-T
P9866	9866	G	0,0031	0,0000	C	CO3	non-syn:T-A
P9887	9887	C	0,0031	0,0000	T	CO3	syn
P9896	9896	G	0,0016	0,0000	A	CO3	syn
P9899	9899	C	0,0078	0,0155	T	CO3	non-syn:F-L
P9903	9903	C	0,0031	0,0077	T	CO3	non-syn:I-M
P9921	9921	A	0,0047	0,0015	G	CO3	syn
P9923	9923	T	0,0016	0,0000	C	CO3	syn
P9932	9932	A	0,0047	0,0077	G	CO3	syn
P9938	9938	C	0,0000	0,0062	T	CO3	non-syn:F-L
P9944	9944	C	0,0016	0,0000	T	CO3	non-syn:A-T
P9947	9947	A	0,0093	0,0015	G	CO3	syn
P9948	9948	A	0,0031	0,0015	G	CO3	syn
P9962	9962	A	0,0078	0,0093	G	CO3	syn
P9966	9966	A	0,0031	0,0015	G	CO3	syn
P9986	9986	A	0,0000	0,0015	G	CO3	syn
P9994	9994	T	0,0016	0,0000	C	CO3	non-syn:V-I
P10003	10003	C	0,0016	0,0000	T	CO3	syn
P10005	10005	G	0,0016	0,0000	A	CO3	non-syn:V-I
P10007	10007	C	0,0000	0,0015	T	CO3	syn

P10031	10031	C	0,0000	0,0015	T	tRNA	-
P10032	10032	G	0,0000	0,0015	A	tRNA	-
P10034	10034	C	0,0202	0,0124	T	tRNA	-
P10042	10042	G	0,0000	0,0046	A	tRNA	-
P10044	10044	G	0,0047	0,0124	A	tRNA	-
P10084	10084	C	0,0031	0,0046	T	tRNA	-
P10086	10086	G	0,0047	0,0031	A	tRNA	-
P10097	10097	G	0,0000	0,0015	A	tRNA	-
P10101	10101	C	0,0062	0,0015	T	tRNA	-
P10115	10115	C	0,0047	0,0000	T	ND3	non-syn:I-T
P10130	10130	C	0,0000	0,0015	A	ND3	non-syn:N-D
P10142	10142	T	0,0031	0,0046	C	ND3	syn
P10154	10154	G	0,0000	0,0015	A	ND3	syn
P10172	10172	A	0,0078	0,0077	G	ND3	syn
P10187	10187	C	0,0000	0,0031	T	ND3	syn
P10188	10188	G	0,0016	0,0000	A	ND3	syn
P10192	10192	T	0,0155	0,0077	C	ND3	syn
P10196	10196	T	0,0016	0,0015	C	ND3	syn
P10202	10202	T	0,0016	0,0000	C	ND3	syn
P10211	10211	T	0,0000	0,0046	C	ND3	non-syn:M-V
P10217	10217	G	0,0000	0,0031	A	ND3	non-syn:S-F
P10237	10237	C	0,0031	0,0062	T	ND3	syn
P10238	10238	C	0,0264	0,0186	T	ND3	syn
P10256	10256	C	0,0031	0,0062	T	ND3	syn
P10262	10262	G	0,0016	0,0000	A	ND3	syn
P10274	10274	C	0,0016	0,0000	T	ND3	non-syn:I-T
P10283	10283	G	0,0062	0,0015	A	ND3	syn
P10289	10289	G	0,0093	0,0093	A	ND3	syn
P10307	10307	T	0,0016	0,0000	C	ND3	syn
P10308	10308	T	0,0031	0,0000	C	ND3	syn
P10310	10310	A	0,0016	0,0000	G	ND3	syn
P10320	10320	A	0,0016	0,0000	G	ND3	syn
P10336	10336	C	0,0016	0,0031	T	ND3	syn:N-N

P10343	10343	T	0,0016	0,0000	C	ND3	syn
P10373	10373	A	0,0016	0,0000	G	ND3	syn
P10376	10376	G	0,0000	0,0015	A	ND3	non-syn:V-I
P10389	10389	C	0,0016	0,0186	T	ND3	non-syn:L-S
P10391	10391	G	0,0016	0,0000	A	ND3	syn
P10394	10394	T	0,0093	0,0015	C	ND3	syn
P10397	10397	G	0,0031	0,0000	A	ND3	syn
P10398	10398	G	0,1690	0,2071	A	ND3	syn
P10399	10399	G	0,0000	0,0015	C	ND3	syn
P10400	10400	T	0,0062	0,0031	C	ND3	syn
P10403	10403	G	0,0016	0,0000	A	ND3	syn
P10410	10410	C	0,0031	0,0077	T	ND3	non-syn:T-A
P10448	10448	C	0,0016	0,0000	T	ND3	non-syn:T-S
P10463	10463	C	0,0977	0,0804	T	ND3	syn
P10465	10465	T	0,0000	0,0015	A	ND3	syn
P10496	10496	G	0,0016	0,0000	A	tRNA	-
P10497	10497	T	0,0016	0,0000	C	tRNA	-
P10499	10499	G	0,0155	0,0232	A	tRNA	-
P10506	10506	G	0,0062	0,0062	A	tRNA	-
P10508	10508	T	0,0000	0,0015	C	ND4L	syn:M-M
P10523	10523	G	0,0016	0,0000	A	ND4L	syn
P10544	10544	T	0,0031	0,0062	C	ND4L	syn
P10550	10550	G	0,0512	0,0804	A	ND4L	non-syn:T-A
P10559	10559	G	0,0031	0,0000	A	ND4L	syn:T-T
P10560	10560	T	0,0016	0,0000	C	ND4L	syn
P10565	10565	T	0,0016	0,0000	C	ND4L	syn
P10586	10586	A	0,0000	0,0031	G	ND4L	syn
P10589	10589	A	0,0109	0,0124	G	ND4L	syn
P10595	10595	C	0,0031	0,0015	T	ND4L	syn
P10598	10598	G	0,0078	0,0031	A	ND4L	syn:C-C
P10609	10609	C	0,0000	0,0031	T	ND4L	syn
P10619	10619	T	0,0016	0,0015	C	ND4L	syn
P10632	10632	C	0,0062	0,0015	T	ND4L	syn

P10644	10644	A	0,0000	0,0015	G	ND4L	syn
P10646	10646	A	0,0016	0,0015	G	ND4L	non-syn:M-T
P10654	10654	T	0,0016	0,0015	C	ND4L	syn
P10658	10658	G	0,0031	0,0015	A	ND4L	syn
P10667	10667	C	0,0016	0,0000	T	ND4L	non-syn:V-M
P10679	10679	G	0,0016	0,0015	A	ND4L	syn
P10680	10680	A	0,0000	0,0015	G	ND4L	non-syn:A-V
P10685	10685	A	0,0047	0,0093	G	ND4L	syn
P10687	10687	G	0,0016	0,0000	T	ND4L	syn
P10688	10688	A	0,0000	0,0015	G	ND4L	syn
P10698	10698	T	0,0016	0,0015	C	ND4L	non-syn:A-T
P10733	10733	T	0,0031	0,0031	C	ND4L	syn
P10742	10742	C	0,0000	0,0015	T	ND4L	non-syn:V-G
P10746	10746	T	0,0000	0,0015	C	ND4L	syn
P10750	10750	G	0,0047	0,0015	A	ND4L	syn
P10771	10771	G	0,0062	0,0062	A	ND4L	syn
P10784	10784	G	0,0016	0,0000	A	ND4L	syn:H-H
P10801	10801	A	0,0000	0,0015	G	ND4L	syn
P10810	10810	C	0,0062	0,0186	T	ND4L	non-syn:N-S
P10819	10819	G	0,0031	0,0031	A	ND4	syn
P10822	10822	T	0,0093	0,0046	C	ND4	non-syn:I-V
P10825	10825	G	0,0047	0,0015	A	ND4	syn
P10861	10861	C	0,0000	0,0031	T	ND4	syn
P10863	10863	A	0,0000	0,0015	G	ND4	syn
P10873	10873	C	0,0078	0,0046	T	ND4	syn
P10876	10876	G	0,0140	0,0108	A	ND4	syn
P10885	10885	C	0,0000	0,0015	T	ND4	syn:I-I
P10900	10900	T	0,0016	0,0000	C	ND4	non-syn:S-N
P10907	10907	C	0,0000	0,0062	T	ND4	syn:P-P
P10909	10909	C	0,0000	0,0015	T	ND4	syn:L-L
P10915	10915	C	0,0078	0,0015	T	ND4	syn
P10922	10922	G	0,0031	0,0015	A	ND4	syn:N-N
P10927	10927	C	0,0140	0,0108	T	ND4	non-syn:F-L

P10961	10961	T	0,0016	0,0000	C	ND4	syn:F-F
P10963	10963	G	0,0000	0,0015	A	ND4	syn
P10966	10966	C	0,0016	0,0000	T	ND4	non-syn:T-A
P10978	10978	G	0,0016	0,0015	A	ND4	syn
P10984	10984	T	0,0000	0,0015	C	ND4	syn:L-L
P10993	10993	A	0,0000	0,0015	G	ND4	syn:L-L
P11002	11002	G	0,0000	0,0031	A	ND4	syn
P11008	11008	T	0,0000	0,0015	C	ND4	syn
P11009	11009	C	0,0000	0,0062	T	ND4	syn
P11016	11016	A	0,0062	0,0031	G	ND4	syn
P11017	11017	C	0,0000	0,0015	T	ND4	syn
P11020	11020	G	0,0016	0,0000	A	ND4	syn
P11024	11024	T	0,0016	0,0000	C	ND4	syn
P11025	11025	C	0,0000	0,0046	T	ND4	non-syn:S-N
P11050	11050	C	0,0016	0,0000	T	ND4	syn
P11065	11065	G	0,0016	0,0000	A	ND4	syn
P11068	11068	G	0,0016	0,0015	A	ND4	syn
P11069	11069	G	0,0000	0,0046	A	ND4	non-syn:L-P
P11071	11071	T	0,0000	0,0015	C	ND4	syn
P11084	11084	G	0,0000	0,0015	A	ND4	syn
P11107	11107	T	0,0016	0,0000	C	ND4	syn
P11128	11128	G	0,0000	0,0015	A	ND4	non-syn:I-V
P11143	11143	T	0,0000	0,0031	C	ND4	syn
P11149	11149	A	0,0016	0,0000	G	ND4	non-syn:T-A
P11150	11150	A	0,0016	0,0000	G	ND4	syn
P11152	11152	C	0,0062	0,0046	T	ND4	syn
P11155	11155	T	0,0000	0,0015	C	ND4	syn
P11167	11167	G	0,0016	0,0015	A	ND4	syn
P11170	11170	T	0,0000	0,0031	C	ND4	non-syn:A-T
P11176	11176	A	0,0031	0,0046	G	ND4	syn
P11191	11191	T	0,0031	0,0062	C	ND4	syn
P11197	11197	T	0,0047	0,0046	C	ND4	syn
P11204	11204	C	0,0031	0,0000	T	ND4	syn:G-G

P11233	11233	C	0,0016	0,0000	T	ND4	syn
P11245	11245	T	0,0000	0,0015	C	ND4	syn
P11251	11251	G	0,1938	0,1978	A	ND4	syn
P11253	11253	C	0,0109	0,0186	T	ND4	non-syn:F-L
P11257	11257	T	0,0016	0,0000	C	ND4	syn
P11260	11260	C	0,0016	0,0000	T	ND4	syn:I-I
P11266	11266	T	0,0016	0,0000	C	ND4	syn
P11287	11287	C	0,0016	0,0000	T	ND4	non-syn:I-T
P11290	11290	G	0,0016	0,0000	A	ND4	syn
P11296	11296	T	0,0000	0,0031	C	ND4	syn
P11299	11299	C	0,0527	0,0788	T	ND4	syn
P11332	11332	T	0,0171	0,0216	C	ND4	syn
P11339	11339	C	0,0016	0,0062	T	ND4	syn:L-L
P11344	11344	G	0,0000	0,0015	A	ND4	syn
P11347	11347	G	0,0016	0,0015	A	ND4	syn
P11353	11353	C	0,0016	0,0015	T	ND4	syn
P11356	11356	T	0,0000	0,0015	C	ND4	syn
P11362	11362	G	0,0016	0,0031	A	ND4	syn
P11365	11365	C	0,0000	0,0015	T	ND4	syn:W-W
P11377	11377	A	0,0233	0,0386	G	ND4	syn
P11386	11386	C	0,0016	0,0000	T	ND4	syn:Y-Y
P11392	11392	G	0,0016	0,0015	A	ND4	syn
P11395	11395	T	0,0000	0,0015	C	ND4	syn
P11410	11410	C	0,0031	0,0000	T	ND4	syn
P11428	11428	T	0,0031	0,0000	C	ND4	syn
P11437	11437	C	0,0000	0,0015	T	ND4	syn:G-G
P11447	11447	A	0,0016	0,0000	G	ND4	syn
P11467	11467	G	0,1798	0,2318	A	ND4	syn
P11470	11470	G	0,0031	0,0015	A	ND4	syn
P11471	11471	T	0,0016	0,0000	C	ND4	syn
P11485	11485	C	0,0078	0,0186	T	ND4	non-syn:V-M
P11548	11548	G	0,0016	0,0000	A	ND4	syn
P11549	11549	T	0,0016	0,0015	C	ND4	syn

P11551	11551	G	0,0000	0,0015	A	ND4	syn
P11560	11560	G	0,0062	0,0015	A	ND4	syn
P11566	11566	G	0,0000	0,0015	A	ND4	syn
P11581	11581	T	0,0031	0,0000	C	ND4	syn
P11582	11582	G	0,0016	0,0000	A	ND4	syn
P11620	11620	G	0,0016	0,0000	A	ND4	syn
P11623	11623	T	0,0000	0,0015	C	ND4	syn
P11653	11653	G	0,0016	0,0031	A	ND4	syn
P11671	11671	G	0,0000	0,0046	A	ND4	non-syn:I-V
P11674	11674	T	0,0248	0,0186	C	ND4	syn
P11696	11696	A	0,0016	0,0000	G	ND4	syn
P11719	11719	A	0,4636	0,5147	G	ND4	syn
P11725	11725	G	0,0016	0,0015	A	ND4	syn
P11732	11732	C	0,0047	0,0046	T	ND4	syn
P11752	11752	G	0,0000	0,0031	C	ND4	non-syn:V-I
P11761	11761	T	0,0000	0,0015	C	ND4	syn:G-G
P11778	11778	A	0,0000	0,0015	G	ND4	syn
P11788	11788	T	0,0000	0,0015	C	ND4	syn
P11794	11794	C	0,0031	0,0015	T	ND4	non-syn:N-K
P11809	11809	C	0,0031	0,0077	T	ND4	syn
P11812	11812	G	0,0853	0,0572	A	ND4	non-syn:R-H
P11830	11830	C	0,0000	0,0015	T	ND4	syn
P11840	11840	T	0,0078	0,0124	C	ND4	syn
P11857	11857	T	0,0016	0,0015	C	ND4	syn
P11860	11860	T	0,0031	0,0000	C	ND4	syn
P11864	11864	C	0,0016	0,0015	T	ND4	syn:F-F
P11869	11869	A	0,0016	0,0046	C	ND4	syn
P11872	11872	T	0,0016	0,0000	C	ND4	syn:N-N
P11875	11875	C	0,0016	0,0000	T	ND4	syn
P11884	11884	G	0,0000	0,0015	A	ND4	syn
P11890	11890	G	0,0000	0,0015	A	ND4	syn
P11893	11893	G	0,0016	0,0015	A	ND4	syn
P11899	11899	C	0,0093	0,0077	T	ND4	syn

P11902	11902	A	0,0000	0,0015	G	ND4	syn:L-L
P11914	11914	A	0,0465	0,0309	G	ND4	syn
P11923	11923	G	0,0062	0,0108	A	ND4	syn
P11929	11929	C	0,0016	0,0000	T	ND4	syn
P11930	11930	G	0,0000	0,0015	A	ND4	syn
P11935	11935	C	0,0031	0,0000	T	ND4	syn
P11936	11936	G	0,0000	0,0015	C	ND4	syn
P11938	11938	T	0,0000	0,0031	C	ND4	syn
P11944	11944	C	0,0047	0,0015	T	ND4	non-syn:I-V
P11947	11947	G	0,0248	0,0186	A	ND4	syn
P11950	11950	G	0,0016	0,0000	A	ND4	non-syn:L-V
P11962	11962	G	0,0016	0,0000	A	ND4	syn
P11969	11969	A	0,0016	0,0000	G	ND4	syn
P11971	11971	T	0,0031	0,0000	C	ND4	syn
P11977	11977	T	0,0016	0,0015	C	ND4	syn
P11989	11989	G	0,0031	0,0031	A	ND4	syn:L-L
P12007	12007	A	0,0047	0,0186	G	ND4	non-syn:A-T
P12017	12017	G	0,0000	0,0015	A	ND4	syn
P12020	12020	T	0,0016	0,0015	C	ND4	syn
P12022	12022	T	0,0016	0,0000	C	ND4	syn
P12026	12026	G	0,0031	0,0000	A	ND4	syn
P12034	12034	T	0,0047	0,0000	C	ND4	non-syn:T-A
P12061	12061	T	0,0016	0,0000	C	ND4	non-syn:H-Y
P12063	12063	T	0,0016	0,0000	C	ND4	syn
P12070	12070	A	0,0000	0,0046	G	ND4	non-syn:I-V
P12083	12083	G	0,0016	0,0046	T	ND4	non-syn:M-I
P12084	12084	T	0,0031	0,0000	C	ND4	syn
P12085	12085	T	0,0016	0,0000	C	ND4	non-syn:T-I
P12127	12127	A	0,0016	0,0015	G	ND4	syn
P12130	12130	C	0,0031	0,0015	T	ND4	non-syn:S-A
P12133	12133	T	0,0016	0,0000	C	ND4	non-syn:S-F
P12135	12135	A	0,0031	0,0015	C	ND4	syn
P12136	12136	C	0,0000	0,0015	T	ND4	syn

P12172	12172	G	0,0031	0,0031	A	ND4	syn
P12192	12192	A	0,0047	0,0000	G	ND4	syn
P12235	12235	C	0,0016	0,0000	T	ND4	non-syn:S-Y
P12236	12236	A	0,0000	0,0015	G	ND4	syn
P12246	12246	T	0,0016	0,0000	C	tRNA	-
P12297	12297	C	0,0000	0,0015	T	tRNA	-
P12308	12308	G	0,1814	0,2287	A	tRNA	-
P12331	12331	G	0,0016	0,0000	A	tRNA	-
P12337	12337	G	0,0000	0,0015	A	tRNA	-
P12341	12341	T	0,0031	0,0000	C	tRNA	-
P12346	12346	T	0,0000	0,0046	C	tRNA	-
P12351	12351	C	0,0016	0,0000	T	tRNA	-
P12358	12358	G	0,0078	0,0031	A	ND5	non-syn:M-V
P12363	12363	T	0,0047	0,0031	C	ND5	non-syn:T-I
P12366	12366	G	0,0000	0,0046	A	ND5	non-syn:H-Y
P12372	12372	A	0,1814	0,2318	G	ND5	syn
P12373	12373	G	0,0000	0,0015	A	ND5	non-syn:T-A
P12376	12376	G	0,0000	0,0015	T	ND5	syn:T-T
P12379	12379	T	0,0000	0,0015	C	ND5	syn
P12384	12384	C	0,0016	0,0000	T	ND5	syn
P12397	12397	G	0,0078	0,0015	A	ND5	non-syn:T-A
P12399	12399	T	0,0000	0,0046	C	ND5	non-syn:S-A
P12403	12403	T	0,0031	0,0031	C	ND5	syn
P12406	12406	A	0,0016	0,0046	G	ND5	syn
P12408	12408	C	0,0016	0,0000	T	ND5	non-syn:T-A
P12414	12414	C	0,0248	0,0201	T	ND5	syn
P12420	12420	G	0,0000	0,0015	A	ND5	non-syn:L-F
P12438	12438	C	0,0000	0,0015	T	ND5	non-syn:V-I
P12441	12441	C	0,0000	0,0031	T	ND5	syn
P12444	12444	G	0,0000	0,0031	A	ND5	syn
P12454	12454	A	0,0016	0,0000	G	ND5	syn
P12471	12471	C	0,0016	0,0000	T	ND5	syn:H-H
P12477	12477	C	0,0000	0,0015	T	ND5	syn

P12481	12481	A	0,0016	0,0000	T	ND5	syn:V-V
P12490	12490	G	0,0000	0,0186	A	ND5	non-syn:V-I
P12491	12491	T	0,0016	0,0000	C	ND5	syn:I-I
P12501	12501	A	0,0264	0,0186	G	ND5	syn:S-S
P12519	12519	C	0,0016	0,0015	T	ND5	non-syn:F-I
P12528	12528	A	0,0000	0,0031	G	ND5	non-syn:T-C
P12537	12537	T	0,0031	0,0000	C	ND5	non-syn:T-M
P12557	12557	T	0,0093	0,0046	C	ND5	syn
P12561	12561	A	0,0016	0,0000	G	ND5	syn
P12562	12562	G	0,0016	0,0062	C	ND5	syn
P12568	12568	T	0,0016	0,0000	C	ND5	syn
P12570	12570	G	0,0000	0,0031	A	ND5	non-syn:T-I
P12582	12582	G	0,0016	0,0000	A	ND5	syn
P12599	12599	C	0,0000	0,0046	T	ND5	non-syn:L-V
P12609	12609	C	0,0000	0,0031	T	ND5	syn
P12612	12612	G	0,0977	0,1206	A	ND5	syn
P12613	12613	A	0,0031	0,0031	G	ND5	syn
P12616	12616	C	0,0062	0,0031	T	ND5	non-syn:M-T
P12618	12618	A	0,0202	0,0232	G	ND5	syn:P-P
P12622	12622	A	0,0000	0,0015	G	ND5	syn:V-V
P12630	12630	A	0,0016	0,0062	G	ND5	non-syn:A-T
P12633	12633	A	0,0109	0,0201	C	ND5	syn:L-L
P12634	12634	G	0,0016	0,0077	A	ND5	syn:L-L
P12636	12636	T	0,0016	0,0000	C	ND5	non-syn:V-I
P12642	12642	G	0,0047	0,0046	A	ND5	syn
P12651	12651	A	0,0047	0,0031	G	ND5	syn
P12654	12654	G	0,0016	0,0186	A	ND5	non-syn:I-V
P12663	12663	A	0,0000	0,0015	C	ND5	syn
P12669	12669	T	0,0031	0,0046	C	ND5	syn
P12674	12674	G	0,0016	0,0000	A	ND5	syn
P12678	12678	C	0,0000	0,0015	T	ND5	syn
P12693	12693	G	0,0016	0,0000	A	ND5	non-syn:N-K
P12705	12705	T	0,0791	0,0711	C	ND5	syn

P12714	12714	C	0,0000	0,0046	T	ND5	non-syn:N-S
P12727	12727	C	0,0000	0,0015	T	ND5	syn:I-I
P12730	12730	A	0,0000	0,0015	G	ND5	syn
P12738	12738	G	0,0000	0,0031	T	ND5	syn
P12741	12741	T	0,0078	0,0015	C	ND5	syn
P12771	12771	A	0,0062	0,0046	G	ND5	syn
P12775	12775	A	0,0000	0,0031	G	ND5	non-syn:V-I
P12795	12795	A	0,0016	0,0000	G	ND5	syn
P12807	12807	G	0,0016	0,0000	A	ND5	syn
P12810	12810	G	0,0016	0,0000	A	ND5	syn:E-E
P12811	12811	C	0,0047	0,0155	T	ND5	non-syn:V-M
P12822	12822	G	0,0016	0,0031	A	ND5	syn
P12834	12834	G	0,0016	0,0015	A	ND5	syn:W-W
P12841	12841	G	0,0000	0,0015	A	ND5	syn
P12842	12842	C	0,0016	0,0000	T	ND5	non-syn:Y-H
P12843	12843	C	0,0016	0,0015	T	ND5	syn
P12855	12855	G	0,0047	0,0015	A	ND5	syn:T-T
P12858	12858	T	0,0000	0,0046	C	ND5	non-syn:I-V
P12879	12879	C	0,0016	0,0031	T	ND5	non-syn:I-T
P12891	12891	T	0,0062	0,0015	C	ND5	syn
P12892	12892	C	0,0000	0,0015	T	ND5	syn
P12923	12923	T	0,0000	0,0015	G	ND5	syn
P12930	12930	G	0,0031	0,0000	A	ND5	syn
P12937	12937	G	0,0047	0,0015	A	ND5	syn:A-A
P12946	12946	T	0,0016	0,0000	C	ND5	syn:L-L
P12950	12950	C	0,0031	0,0031	A	ND5	non-syn:W-L
P12954	12954	C	0,0000	0,0031	T	ND5	syn
P12957	12957	C	0,0047	0,0000	T	ND5	non-syn:M-V
P12975	12975	G	0,0000	0,0015	A	ND5	syn:L-L
P13020	13020	C	0,0217	0,0124	T	ND5	non-syn:N-T
P13035	13035	A	0,0016	0,0000	C	ND5	syn
P13047	13047	G	0,0016	0,0000	A	ND5	syn
P13050	13050	G	0,0016	0,0000	A	ND5	syn:L-L

P13056	13056	T	0,0000	0,0015	C	ND5	syn
P13101	13101	C	0,0016	0,0015	A	ND5	syn:L-L
P13104	13104	G	0,0000	0,0031	A	ND5	syn:M-M
P13105	13105	G	0,0109	0,0077	A	ND5	syn
P13111	13111	C	0,0000	0,0015	T	ND5	syn
P13117	13117	G	0,0016	0,0000	A	ND5	syn
P13135	13135	A	0,0016	0,0108	G	ND5	syn
P13145	13145	A	0,0093	0,0186	G	ND5	non-syn:I-V
P13152	13152	T	0,0016	0,0000	A	ND5	syn
P13158	13158	G	0,0000	0,0015	A	ND5	syn
P13161	13161	C	0,0031	0,0000	T	ND5	non-syn:A-T
P13188	13188	T	0,0016	0,0046	C	ND5	non-syn:S-N
P13191	13191	G	0,0016	0,0000	T	ND5	syn
P13194	13194	A	0,0000	0,0015	G	ND5	syn
P13200	13200	G	0,0000	0,0015	A	ND5	syn
P13203	13203	G	0,0016	0,0000	A	ND5	syn
P13204	13204	A	0,0000	0,0015	G	ND5	syn
P13209	13209	T	0,0031	0,0031	C	ND5	syn:L-L
P13224	13224	C	0,0016	0,0000	T	ND5	syn
P13263	13263	G	0,0000	0,0015	A	ND5	syn:A-A
P13269	13269	G	0,0016	0,0000	A	ND5	non-syn:V-I
P13281	13281	C	0,0047	0,0015	T	ND5	syn:C-C
P13287	13287	T	0,0016	0,0000	C	ND5	syn:N-N
P13293	13293	T	0,0016	0,0031	C	ND5	syn:Q-Q
P13317	13317	A	0,0031	0,0015	G	ND5	syn:G-G
P13323	13323	T	0,0016	0,0031	C	ND5	syn
P13326	13326	C	0,0078	0,0077	T	ND5	syn
P13327	13327	G	0,0016	0,0000	A	ND5	syn
P13347	13347	T	0,0016	0,0000	C	ND5	syn
P13350	13350	C	0,0016	0,0000	A	ND5	syn
P13356	13356	C	0,0000	0,0015	T	ND5	syn
P13359	13359	A	0,0000	0,0046	G	ND5	non-syn:T-A
P13362	13362	T	0,0031	0,0031	C	ND5	syn:A-A

P13368	13368	A	0,0961	0,0773	G	ND5	non-syn:M-I
P13395	13395	G	0,0062	0,0046	A	ND5	syn
P13401	13401	C	0,0016	0,0062	T	ND5	syn
P13413	13413	G	0,0016	0,0000	A	ND5	syn:C-C
P13419	13419	G	0,0016	0,0000	A	ND5	syn
P13434	13434	G	0,0000	0,0062	A	ND5	syn
P13470	13470	G	0,0016	0,0000	A	ND5	syn
P13500	13500	C	0,0078	0,0124	T	ND5	syn
P13506	13506	T	0,0000	0,0015	C	ND5	syn
P13525	13525	A	0,0000	0,0015	G	ND5	syn
P13528	13528	G	0,0016	0,0015	A	ND5	syn:L-L
P13542	13542	G	0,0016	0,0000	A	ND5	syn
P13557	13557	G	0,0031	0,0046	A	ND5	syn
P13564	13564	C	0,0016	0,0000	T	ND5	non-syn:E-K
P13565	13565	T	0,0016	0,0046	C	ND5	non-syn:T-A
P13575	13575	T	0,0016	0,0000	C	ND5	syn
P13578	13578	T	0,0016	0,0000	C	ND5	syn
P13581	13581	C	0,0016	0,0031	T	ND5	non-syn:S-P
P13590	13590	A	0,0078	0,0015	G	ND5	non-syn:S-F
P13602	13602	C	0,0000	0,0031	T	ND5	syn
P13617	13617	C	0,0729	0,0989	T	ND5	syn
P13620	13620	C	0,0000	0,0015	T	ND5	syn:A-A
P13627	13627	T	0,0000	0,0046	C	ND5	syn
P13630	13630	G	0,0016	0,0031	A	ND5	syn
P13632	13632	G	0,0000	0,0015	A	ND5	syn
P13635	13635	C	0,0016	0,0031	T	ND5	syn
P13637	13637	G	0,0109	0,0155	A	ND5	syn:L-L
P13650	13650	T	0,0031	0,0093	C	ND5	non-syn:T-A
P13651	13651	G	0,0000	0,0015	A	ND5	syn:T-T
P13656	13656	C	0,0016	0,0000	T	ND5	syn
P13680	13680	T	0,0078	0,0093	C	ND5	non-syn:Q-R
P13681	13681	G	0,0031	0,0015	A	ND5	syn
P13692	13692	T	0,0016	0,0015	C	ND5	non-syn:T-A

P13704	13704	T	0,0000	0,0015	C	ND5	syn
P13705	13705	T	0,0000	0,0015	C	ND5	syn
P13708	13708	A	0,1101	0,1329	G	ND5	non-syn:T-A
P13710	13710	G	0,0000	0,0015	A	ND5	syn
P13711	13711	A	0,0016	0,0015	G	ND5	syn
P13713	13713	G	0,0016	0,0000	C	ND5	syn
P13720	13720	T	0,0000	0,0015	C	ND5	non-syn:A-T
P13722	13722	G	0,0171	0,0201	A	ND5	syn
P13734	13734	C	0,0140	0,0093	T	ND5	non-syn:A-T
P13740	13740	C	0,0078	0,0124	T	ND5	syn
P13743	13743	C	0,0016	0,0031	T	ND5	syn
P13752	13752	C	0,0016	0,0077	T	ND5	syn
P13759	13759	A	0,0202	0,0155	G	ND5	syn
P13762	13762	G	0,0031	0,0015	T	ND5	syn
P13768	13768	C	0,0000	0,0015	T	ND5	syn
P13780	13780	G	0,0233	0,0139	A	ND5	syn
P13785	13785	T	0,0000	0,0031	C	ND5	non-syn:A-T
P13789	13789	C	0,0000	0,0015	T	ND5	non-syn:S-A
P13803	13803	G	0,0031	0,0000	A	ND5	non-syn:F-L
P13827	13827	G	0,0016	0,0124	A	ND5	non-syn:I-V
P13830	13830	C	0,0016	0,0000	T	ND5	syn
P13834	13834	G	0,0016	0,0046	A	ND5	non-syn:Y-H
P13840	13840	T	0,0016	0,0000	C	ND5	syn:T-T
P13854	13854	T	0,0000	0,0015	C	ND5	syn
P13880	13880	A	0,0000	0,0015	C	ND5	syn
P13899	13899	C	0,0000	0,0031	T	ND5	non-syn:T-A
P13923	13923	T	0,0031	0,0015	C	ND5	syn
P13928	13928	C	0,0016	0,0108	G	ND5	syn
P13933	13933	G	0,0047	0,0015	A	ND5	non-syn:S-Y
P13934	13934	T	0,0171	0,0139	C	ND5	syn
P13942	13942	G	0,0000	0,0015	A	ND5	syn
P13943	13943	T	0,0016	0,0046	C	ND5	non-syn:S-T
P13948	13948	T	0,0016	0,0015	C	ND5	non-syn:T-A

P13953	13953	C	0,0016	0,0000	T	ND5	non-syn:T-M
P13958	13958	C	0,0000	0,0015	G	ND5	non-syn:T-A
P13959	13959	T	0,0016	0,0046	C	ND5	non-syn:T-M
P13965	13965	C	0,0171	0,0108	T	ND5	non-syn:P-S
P13966	13966	G	0,0295	0,0263	A	ND5	syn
P13967	13967	T	0,0062	0,0186	C	ND5	non-syn:G-A
P13980	13980	A	0,0016	0,0000	G	ND5	syn
P14002	14002	G	0,0031	0,0046	A	ND5	syn
P14016	14016	A	0,0000	0,0031	G	ND5	non-syn:T-A
P14020	14020	C	0,0000	0,0015	T	ND5	non-syn:T-M
P14034	14034	C	0,0000	0,0015	T	ND5	syn
P14037	14037	G	0,0016	0,0046	A	ND5	non-syn:T-A
P14040	14040	A	0,0047	0,0046	G	ND5	syn
P14053	14053	G	0,0000	0,0031	A	ND5	syn
P14061	14061	T	0,0000	0,0015	C	ND5	syn
P14062	14062	G	0,0000	0,0062	A	ND5	syn
P14070	14070	G	0,0000	0,0031	A	ND5	syn
P14071	14071	G	0,0000	0,0015	A	ND5	non-syn:T-A
P14097	14097	T	0,0016	0,0000	C	ND5	syn:I-I
P14110	14110	C	0,0031	0,0046	T	ND5	non-syn:I-V
P14118	14118	G	0,0031	0,0031	A	ND5	syn:S-S
P14133	14133	G	0,0155	0,0201	A	ND5	non-syn:T-A
P14139	14139	G	0,0078	0,0077	A	ND5	syn:Y-Y
P14148	14148	G	0,0078	0,0031	A	ND5	non-syn:F-L
P14149	14149	T	0,0016	0,0000	C	ND5	syn:P-P
P14163	14163	T	0,0000	0,0015	C	ND5	syn:L-L
P14167	14167	T	0,0574	0,0819	C	ND5	syn:L-L
P14178	14178	C	0,0000	0,0015	T	ND5	syn:TERM-TERM
P14179	14179	G	0,0047	0,0015	A	ND6	syn:TERM-TERM
P14182	14182	C	0,0388	0,0371	T	ND6	non-syn:A-T
P14194	14194	T	0,0016	0,0000	C	ND6	syn:E-E
P14198	14198	A	0,0016	0,0000	G	ND6	non-syn:I-V
P14203	14203	G	0,0047	0,0031	A	ND6	syn

P14212	14212	C	0,0078	0,0015	T	ND6	syn
P14220	14220	G	0,0016	0,0015	A	ND6	syn
P14221	14221	C	0,0000	0,0015	T	ND6	non-syn:T-M
P14233	14233	G	0,0853	0,0572	A	ND6	syn
P14249	14249	A	0,0031	0,0000	G	ND6	syn:V-V
P14258	14258	A	0,0016	0,0015	G	ND6	syn
P14260	14260	G	0,0000	0,0031	A	ND6	syn
P14274	14274	G	0,0016	0,0000	A	ND6	syn
P14290	14290	C	0,0000	0,0015	T	ND6	non-syn:A-V
P14299	14299	C	0,0000	0,0015	T	ND6	non-syn:P-L
P14305	14305	A	0,0047	0,0093	G	ND6	syn
P14311	14311	C	0,0000	0,0015	T	ND6	syn:L-L
P14318	14318	C	0,0000	0,0015	T	ND6	syn:E-E
P14319	14319	C	0,0031	0,0015	T	ND6	syn
P14323	14323	A	0,0000	0,0077	G	ND6	syn
P14325	14325	C	0,0016	0,0031	T	ND6	syn:V-V
P14364	14364	A	0,0047	0,0062	G	ND6	non-syn:N-S
P14365	14365	T	0,0109	0,0186	C	ND6	non-syn:N-D
P14394	14394	T	0,0016	0,0000	C	ND6	syn
P14405	14405	G	0,0016	0,0015	A	ND6	non-syn:N-D
P14434	14434	T	0,0000	0,0015	C	ND6	syn
P14437	14437	G	0,0031	0,0015	A	ND6	syn
P14446	14446	T	0,0016	0,0000	C	ND6	non-syn:V-M
P14476	14476	A	0,0016	0,0046	G	ND6	non-syn:V-A
P14484	14484	C	0,0000	0,0015	T	ND6	syn:E-E
P14488	14488	C	0,0016	0,0000	T	ND6	syn
P14502	14502	C	0,0047	0,0031	T	ND6	syn:E-E
P14518	14518	G	0,0016	0,0031	A	ND6	syn
P14550	14550	C	0,0000	0,0031	T	ND6	non-syn:M-V
P14552	14552	G	0,0016	0,0015	A	ND6	syn
P14560	14560	A	0,0000	0,0031	G	ND6	non-syn:I-V
P14562	14562	T	0,0016	0,0000	C	ND6	syn
P14566	14566	G	0,0031	0,0000	A	ND6	non-syn:I-V

P14569	14569	A	0,0109	0,0186	G	ND6	non-syn:V-A
P14577	14577	G	0,0016	0,0000	T	ND6	syn
P14581	14581	C	0,0031	0,0000	T	ND6	non-syn:V-I
P14582	14582	G	0,0124	0,0186	A	ND6	syn:G=-G
P14587	14587	G	0,0047	0,0031	A	ND6	syn
P14605	14605	G	0,0016	0,0015	A	ND6	non-syn:I-L
P14620	14620	T	0,0171	0,0232	C	ND6	syn:V-V
P14632	14632	T	0,0016	0,0015	C	ND6	non-syn:V-A
P14634	14634	C	0,0000	0,0015	T	ND6	syn:G-G
P14647	14647	G	0,0031	0,0000	A	ND6	syn
P14655	14655	T	0,0016	0,0000	G	ND6	syn
P14668	14668	G	0,0016	0,0000	C	ND6	syn
P14684	14684	T	0,0000	0,0015	C	ND6	non-syn:M-V
P14687	14687	G	0,0124	0,0093	A	ND6	non-syn:T-A
P14696	14696	G	0,0016	0,0000	A	ND6	non-syn:L-M
P14727	14727	C	0,0016	0,0000	T	ND6	non-syn:H-D
P14743	14743	C	0,0000	0,0015	A	tRNA	-
P14745	14745	T	0,0031	0,0000	C	tRNA	-
P14750	14750	G	0,0000	0,0015	A	tRNA	-
P14766	14766	T	0,4496	0,5085	C	tRNA	-
P14769	14769	G	0,0000	0,0015	A	Non-Coding	-
P14770	14770	T	0,0062	0,0000	C	Non-Coding	-
P14783	14783	C	0,0047	0,0031	T	CYB	non-syn:T-A
P14793	14793	G	0,0372	0,0788	A	CYB	non-syn:T-I
P14798	14798	C	0,1194	0,1530	T	CYB	non-syn:N-S
P14831	14831	A	0,0062	0,0015	G	CYB	syn
P14839	14839	G	0,0016	0,0000	A	CYB	syn
P14854	14854	A	0,0000	0,0015	C	CYB	non-syn:H-R
P14856	14856	C	0,0000	0,0015	T	CYB	non-syn:F-L
P14857	14857	C	0,0016	0,0046	T	CYB	non-syn:A-T
P14863	14863	T	0,0000	0,0015	C	CYB	syn
P14866	14866	T	0,0000	0,0062	C	CYB	syn
P14869	14869	A	0,0016	0,0046	G	CYB	non-syn:L-P

P14870	14870	G	0,0016	0,0000	A	CYB	syn
P14871	14871	C	0,0016	0,0000	T	CYB	syn:A-A
P14872	14872	T	0,0109	0,0139	C	CYB	syn
P14893	14893	G	0,0016	0,0000	A	CYB	syn
P14902	14902	T	0,0016	0,0046	C	CYB	non-syn:I-V
P14905	14905	A	0,0946	0,0773	G	CYB	non-syn:I-T
P14927	14927	G	0,0016	0,0015	A	CYB	syn
P14953	14953	T	0,0031	0,0000	C	CYB	syn
P14954	14954	G	0,0000	0,0015	A	CYB	syn
P14956	14956	C	0,0031	0,0015	T	CYB	syn
P14968	14968	C	0,0016	0,0093	T	CYB	non-syn:T-A
P14971	14971	C	0,0047	0,0015	T	CYB	syn
P14979	14979	C	0,0016	0,0000	T	CYB	non-syn:T-A
P14983	14983	T	0,0000	0,0015	C	CYB	syn
P15010	15010	G	0,0016	0,0000	A	CYB	syn
P15013	15013	G	0,0000	0,0015	A	CYB	syn
P15014	15014	C	0,0016	0,0000	T	CYB	non-syn:I-T
P15016	15016	T	0,0016	0,0000	C	CYB	syn
P15019	15019	C	0,0000	0,0015	T	CYB	syn
P15028	15028	A	0,0031	0,0077	C	CYB	syn:M-M
P15043	15043	A	0,0295	0,0247	G	CYB	non-syn:F-L
P15047	15047	A	0,0000	0,0015	G	CYB	syn
P15058	15058	T	0,0031	0,0000	C	CYB	syn:F-F
P15067	15067	C	0,0016	0,0015	T	CYB	syn
P15077	15077	A	0,0016	0,0000	G	CYB	syn
P15088	15088	T	0,0000	0,0015	C	CYB	non-syn:G-S
P15106	15106	A	0,0000	0,0031	G	CYB	syn
P15110	15110	A	0,0016	0,0015	G	CYB	syn
P15115	15115	C	0,0000	0,0046	T	CYB	non-syn:E-K
P15133	15133	G	0,0000	0,0015	A	CYB	syn
P15148	15148	A	0,0000	0,0077	G	CYB	syn
P15159	15159	C	0,0031	0,0000	T	CYB	non-syn:A-T
P15164	15164	C	0,0016	0,0000	T	CYB	syn:T-T

P15172	15172	A	0,0000	0,0031	G	CYB	syn
P15178	15178	G	0,0016	0,0000	A	CYB	syn
P15191	15191	C	0,0031	0,0046	T	CYB	non-syn:M-T
P15199	15199	A	0,0000	0,0046	C	CYB	non-syn:F-L
P15202	15202	T	0,0000	0,0015	C	CYB	syn
P15213	15213	C	0,0000	0,0015	T	CYB	syn
P15217	15217	A	0,0047	0,0031	G	CYB	syn:L-L
P15218	15218	G	0,0310	0,0386	A	CYB	syn
P15226	15226	G	0,0000	0,0015	A	CYB	syn
P15244	15244	G	0,0016	0,0046	A	CYB	non-syn:I-T
P15247	15247	G	0,0000	0,0015	C	CYB	syn
P15257	15257	A	0,0279	0,0433	G	CYB	non-syn:T-A
P15262	15262	C	0,0016	0,0000	T	CYB	syn:L-L
P15287	15287	C	0,0000	0,0031	T	CYB	syn
P15289	15289	C	0,0000	0,0015	T	CYB	syn
P15299	15299	C	0,0000	0,0015	T	CYB	non-syn:D-N
P15301	15301	A	0,0078	0,0077	G	CYB	syn
P15310	15310	C	0,0031	0,0000	T	CYB	non-syn:F-L
P15313	15313	C	0,0000	0,0015	T	CYB	syn:F-F
P15314	15314	A	0,0016	0,0015	G	CYB	syn:L-L
P15315	15315	T	0,0016	0,0000	C	CYB	syn
P15316	15316	G	0,0000	0,0015	A	CYB	syn
P15326	15326	A	0,0326	0,0093	G	CYB	syn
P15340	15340	G	0,0000	0,0046	A	CYB	non-syn:A-T
P15346	15346	A	0,0016	0,0000	G	CYB	non-syn:A-V
P15355	15355	A	0,0047	0,0000	G	CYB	syn:A-A
P15367	15367	T	0,0016	0,0000	C	CYB	non-syn:T-A
P15373	15373	G	0,0016	0,0000	A	CYB	syn
P15381	15381	T	0,0016	0,0000	C	CYB	syn
P15400	15400	T	0,0000	0,0015	C	CYB	syn
P15409	15409	T	0,0031	0,0000	C	CYB	syn
P15440	15440	C	0,0000	0,0015	T	CYB	syn
P15448	15448	A	0,0000	0,0031	C	CYB	non-syn:T-I

P15449	15449	C	0,0000	0,0015	T	CYB	syn
P15450	15450	C	0,0000	0,0015	T	CYB	syn
P15452	15452	A	0,1938	0,1963	C	CYB	syn
P15454	15454	C	0,0093	0,0108	T	CYB	syn:L-L
P15456	15456	G	0,0016	0,0046	T	CYB	non-syn:F-L
P15457	15457	T	0,0016	0,0000	C	CYB	non-syn:F-S
P15459	15459	T	0,0016	0,0000	C	CYB	non-syn:L-I
P15466	15466	A	0,0031	0,0031	G	CYB	syn
P15467	15467	G	0,0016	0,0000	A	CYB	non-syn:L-R
P15470	15470	C	0,0016	0,0000	T	CYB	syn:L-L
P15479	15479	C	0,0031	0,0046	T	CYB	non-syn:S-F
P15497	15497	A	0,0016	0,0046	G	CYB	syn
P15498	15498	A	0,0016	0,0046	G	CYB	non-syn:T-A
P15506	15506	A	0,0000	0,0015	G	CYB	syn
P15511	15511	C	0,0047	0,0046	T	CYB	non-syn:F-L
P15519	15519	C	0,0016	0,0000	T	CYB	non-syn:G-S
P15530	15530	C	0,0000	0,0015	T	CYB	non-syn:G-D
P15541	15541	C	0,0016	0,0015	T	CYB	non-syn:D-N
P15542	15542	T	0,0016	0,0015	C	CYB	syn
P15547	15547	T	0,0000	0,0046	C	CYB	non-syn:L-P
P15553	15553	A	0,0000	0,0015	G	CYB	syn
P15607	15607	G	0,0946	0,0773	A	CYB	syn:P-P
P15613	15613	G	0,0000	0,0046	A	CYB	non-syn:P-S
P15616	15616	T	0,0016	0,0000	C	CYB	syn
P15617	15617	A	0,0016	0,0000	G	CYB	syn
P15618	15618	C	0,0000	0,0015	T	CYB	syn
P15630	15630	C	0,0016	0,0000	T	CYB	syn
P15632	15632	T	0,0000	0,0015	C	CYB	syn:G-G
P15645	15645	C	0,0016	0,0000	T	CYB	non-syn:V-I
P15646	15646	T	0,0000	0,0015	C	CYB	non-syn:V-A
P15657	15657	C	0,0000	0,0046	T	CYB	non-syn:L-S
P15670	15670	C	0,0016	0,0015	T	CYB	syn:L-L
P15672	15672	C	0,0000	0,0015	T	CYB	non-syn:I-T

P15679	15679	G	0,0000	0,0031	A	CYB	syn
P15693	15693	C	0,0171	0,0232	T	CYB	non-syn:I-T
P15697	15697	C	0,0016	0,0000	T	CYB	syn:H-H
P15713	15713	G	0,0016	0,0000	T	CYB	non-syn:M-T
P15715	15715	G	0,0031	0,0015	A	CYB	syn
P15721	15721	C	0,0031	0,0015	T	CYB	non-syn:M-T
P15734	15734	A	0,0016	0,0015	G	CYB	syn
P15742	15742	A	0,0000	0,0031	C	CYB	non-syn:S-A
P15758	15758	G	0,0093	0,0077	A	CYB	syn
P15773	15773	A	0,0016	0,0031	G	CYB	syn
P15775	15775	G	0,0016	0,0031	A	CYB	non-syn:A-T
P15784	15784	C	0,0016	0,0015	T	CYB	syn
P15789	15789	T	0,0000	0,0046	C	CYB	non-syn:I-V
P15790	15790	T	0,0000	0,0031	C	CYB	non-syn:V-M
P15804	15804	C	0,0016	0,0046	T	CYB	syn
P15812	15812	A	0,0093	0,0077	G	CYB	syn
P15813	15813	C	0,0000	0,0046	T	CYB	non-syn:T-I
P15815	15815	T	0,0000	0,0015	C	CYB	syn
P15817	15817	G	0,0000	0,0046	A	CYB	non-syn:V-A
P15833	15833	T	0,0124	0,0046	C	CYB	non-syn:V-M
P15852	15852	C	0,0000	0,0046	T	CYB	non-syn:V-A
P15883	15883	A	0,0000	0,0046	G	CYB	syn:L-L
P15891	15891	T	0,0000	0,0015	C	CYB	syn
P15894	15894	A	0,0000	0,0015	G	CYB	syn
P15901	15901	G	0,0016	0,0000	A	CYB	non-syn:I-T
P15903	15903	G	0,0000	0,0015	A	CYB	syn
P15904	15904	T	0,0527	0,0510	C	tRNA	-
P15907	15907	G	0,0140	0,0093	A	tRNA	-
P15910	15910	T	0,0016	0,0000	C	tRNA	-
P15911	15911	T	0,0016	0,0046	A	tRNA	-
P15913	15913	T	0,0016	0,0000	C	tRNA	-
P15917	15917	T	0,0000	0,0015	C	tRNA	-
P15924	15924	G	0,0419	0,0263	A	tRNA	-

P15927	15927	A	0,0109	0,0046	G	tRNA	-
P15928	15928	A	0,1023	0,0773	G	tRNA	-
P15930	15930	A	0,0016	0,0015	G	tRNA	-
P15941	15941	C	0,0047	0,0000	T	tRNA	-
P15946	15946	T	0,0078	0,0077	C	tRNA	-
P15947	15947	G	0,0000	0,0015	A	tRNA	-
P15954	15954	C	0,0000	0,0031	A	tRNA	-
P15968	15968	C	0,0016	0,0000	T	tRNA	-
P15983	15983	C	0,0016	0,0000	T	tRNA	-
P15992	15992	G	0,0000	0,0015	A	tRNA	-
P16037	16037	G	0,0031	0,0093	A	Non-Coding	-
P16051	16051	G	0,0233	0,0139	A	tRNA	-
P16067	16067	T	0,0000	0,0015	C	tRNA	-
P16069	16069	T	0,0992	0,1221	C	tRNA	-
P16070	16070	G	0,0000	0,0015	A	Control-Region	-
P16071	16071	T	0,0000	0,0062	C	Control-Region	-
P16074	16074	G	0,0016	0,0000	A	Control-Region	-
P16075	16075	C	0,0016	0,0015	T	Control-Region	-
P16080	16080	G	0,0031	0,0000	A	Control-Region	-
P16086	16086	C	0,0078	0,0093	T	Control-Region	-
P16092	16092	C	0,0078	0,0077	T	Control-Region	-
P16093	16093	C	0,0605	0,0712	T	Control-Region	-
P16104	16104	T	0,0031	0,0000	C	Control-Region	-
P16108	16108	T	0,0000	0,0031	C	Control-Region	-
P16124	16124	C	0,0031	0,0015	T	Control-Region	-
P16126	16126	C	0,2031	0,1978	T	Control-Region	-
P16127	16127	G	0,0000	0,0015	A	Control-Region	-
P16134	16134	T	0,0047	0,0031	C	Control-Region	-
P16136	16136	C	0,0031	0,0000	T	Control-Region	-
P16140	16140	C	0,0047	0,0031	T	Control-Region	-
P16144	16144	C	0,0171	0,0186	T	Control-Region	-
P16145	16145	A	0,0295	0,0495	G	Control-Region	-
P16146	16146	G	0,0016	0,0000	A	Control-Region	-

P16148	16148	T	0,0062	0,0015	C	Control-Region	-
P16150	16150	T	0,0031	0,0015	C	Control-Region	-
P16153	16153	A	0,0140	0,0108	G	Control-Region	-
P16157	16157	C	0,0016	0,0000	T	Control-Region	-
P16158	16158	G	0,0016	0,0000	A	Control-Region	-
P16162	16162	G	0,0357	0,0340	A	Control-Region	-
P16163	16163	G	0,0233	0,0216	A	Control-Region	-
P16167	16167	T	0,0000	0,0015	C	Control-Region	-
P16168	16168	T	0,0047	0,0031	C	Control-Region	-
P16169	16169	T	0,0016	0,0000	C	Control-Region	-
P16170	16170	G	0,0000	0,0015	A	Control-Region	-
P16171	16171	G	0,0031	0,0015	A	Control-Region	-
P16172	16172	C	0,0295	0,0510	T	Control-Region	-
P16173	16173	T	0,0031	0,0000	C	Control-Region	-
P16177	16177	G	0,0016	0,0000	A	Control-Region	-
P16179	16179	T	0,0000	0,0062	C	Control-Region	-
P16180	16180	T	0,0016	0,0000	A	Control-Region	-
P16181	16181	G	0,0016	0,0000	A	Control-Region	-
P16182	16182	C	0,0171	0,0186	A	Control-Region	-
P16185	16185	T	0,0031	0,0015	C	Control-Region	-
P16186	16186	T	0,0124	0,0216	C	Control-Region	-
P16187	16187	T	0,0031	0,0031	C	Control-Region	-
P16188	16188	T	0,0031	0,0000	C	Control-Region	-
P16189	16189	C	0,1630	0,1376	T	Control-Region	-
P16192	16192	T	0,0357	0,0649	C	Control-Region	-
P16194	16194	C	0,0016	0,0000	A	Control-Region	-
P16195	16195	C	0,0016	0,0000	T	Control-Region	-
P16201	16201	T	0,0000	0,0015	C	Control-Region	-
P16203	16203	G	0,0000	0,0046	A	Control-Region	-
P16209	16209	C	0,0140	0,0232	T	Control-Region	-
P16213	16213	A	0,0093	0,0186	G	Control-Region	-
P16215	16215	G	0,0016	0,0015	A	Control-Region	-
P16217	16217	C	0,0016	0,0046	T	Control-Region	-

P16218	16218	T	0,0031	0,0062	C	Control-Region	-
P16219	16219	G	0,0078	0,0046	A	Control-Region	-
P16221	16221	T	0,0031	0,0031	C	Control-Region	-
P16222	16222	T	0,0093	0,0186	C	Control-Region	-
P16223	16223	T	0,0744	0,0757	C	Control-Region	-
P16224	16224	C	0,0543	0,0757	T	Control-Region	-
P16231	16231	C	0,0155	0,0216	T	Control-Region	-
P16234	16234	T	0,0093	0,0077	C	Control-Region	-
P16235	16235	G	0,0031	0,0046	A	Control-Region	-
P16239	16239	T	0,0047	0,0077	C	Control-Region	-
P16241	16241	G	0,0000	0,0015	A	Control-Region	-
P16243	16243	C	0,0047	0,0031	T	Control-Region	-
P16245	16245	T	0,0062	0,0124	C	Control-Region	-
P16246	16246	T	0,0000	0,0015	A	Control-Region	-
P16247	16247	G	0,0016	0,0000	A	Control-Region	-
P16248	16248	T	0,0078	0,0000	C	Control-Region	-
P16249	16249	C	0,0062	0,0124	T	Control-Region	-
P16253	16253	T	0,0000	0,0031	A	Control-Region	-
P16254	16254	G	0,0016	0,0000	A	Control-Region	-
P16255	16255	A	0,0031	0,0139	G	Control-Region	-
P16256	16256	T	0,0543	0,0697	C	Control-Region	-
P16257	16257	T	0,0016	0,0077	C	Control-Region	-
P16259	16259	T	0,0000	0,0015	C	Control-Region	-
P16260	16260	T	0,0016	0,0015	C	Control-Region	-
P16262	16262	T	0,0000	0,0015	C	Control-Region	-
P16263	16263	C	0,0155	0,0201	T	Control-Region	-
P16264	16264	T	0,0000	0,0031	C	Control-Region	-
P16265	16265	G	0,0031	0,0031	A	Control-Region	-
P16266	16266	T	0,0093	0,0062	C	Control-Region	-
P16271	16271	C	0,0016	0,0062	T	Control-Region	-
P16274	16274	A	0,0078	0,0062	G	Control-Region	-
P16278	16278	T	0,0357	0,0356	C	Control-Region	-
P16283	16283	G	0,0000	0,0046	A	Control-Region	-

P16284	16284	G	0,0016	0,0000	A	Control-Region	-
P16287	16287	T	0,0047	0,0031	C	Control-Region	-
P16290	16290	T	0,0016	0,0093	C	Control-Region	-
P16291	16291	T	0,0171	0,0232	C	Control-Region	-
P16292	16292	T	0,0310	0,0263	C	Control-Region	-
P16294	16294	T	0,1101	0,1005	C	Control-Region	-
P16295	16295	T	0,0062	0,0031	C	Control-Region	-
P16296	16296	T	0,0574	0,0325	C	Control-Region	-
P16297	16297	C	0,0016	0,0046	T	Control-Region	-
P16299	16299	G	0,0047	0,0062	A	Control-Region	-
P16300	16300	G	0,0016	0,0015	A	Control-Region	-
P16301	16301	T	0,0062	0,0062	C	Control-Region	-
P16302	16302	G	0,0000	0,0015	A	Control-Region	-
P16304	16304	C	0,0729	0,0510	T	Control-Region	-
P16305	16305	G	0,0000	0,0015	A	Control-Region	-
P16309	16309	G	0,0031	0,0077	A	Control-Region	-
P16310	16310	A	0,0016	0,0000	G	Control-Region	-
P16311	16311	C	0,1273	0,1546	T	Control-Region	-
P16316	16316	G	0,0093	0,0046	A	Control-Region	-
P16318	16318	T	0,0031	0,0031	A	Control-Region	-
P16319	16319	A	0,0109	0,0216	G	Control-Region	-
P16320	16320	T	0,0155	0,0170	C	Control-Region	-
P16322	16322	G	0,0016	0,0000	A	Control-Region	-
P16324	16324	C	0,0124	0,0062	T	Control-Region	-
P16325	16325	C	0,0155	0,0170	T	Control-Region	-
P16327	16327	T	0,0000	0,0031	C	Control-Region	-
P16328	16328	A	0,0000	0,0046	C	Control-Region	-
P16335	16335	G	0,0031	0,0015	A	Control-Region	-
P16342	16342	C	0,0047	0,0031	T	Control-Region	-
P16344	16344	T	0,0031	0,0015	C	Control-Region	-
P16350	16350	G	0,0000	0,0031	A	Control-Region	-
P16352	16352	C	0,0016	0,0031	T	Control-Region	-
P16354	16354	T	0,0217	0,0186	C	Control-Region	-

P16355	16355	T	0,0124	0,0108	C	Control-Region	-
P16356	16356	C	0,0279	0,0340	T	Control-Region	-
P16357	16357	C	0,0016	0,0015	T	Control-Region	-
P16359	16359	C	0,0000	0,0015	T	Control-Region	-
P16360	16360	T	0,0031	0,0000	C	Control-Region	-
P16362	16362	C	0,0636	0,0726	T	Control-Region	-
P16365	16365	T	0,0031	0,0046	C	Control-Region	-
P16366	16366	T	0,0016	0,0015	C	Control-Region	-
P16368	16368	C	0,0016	0,0000	T	Control-Region	-
P16390	16390	A	0,0140	0,0170	G	Control-Region	-
P16391	16391	A	0,0202	0,0139	G	Control-Region	-
P16398	16398	A	0,0031	0,0031	G	Control-Region	-
P16399	16399	G	0,0341	0,0402	A	Control-Region	-
P16400	16400	T	0,0124	0,0093	C	Control-Region	-
P16428	16428	A	0,0016	0,0015	G	Control-Region	-
P16438	16438	A	0,0031	0,0015	G	Control-Region	-
P16463	16463	G	0,0047	0,0077	A	Control-Region	-
P16465	16465	T	0,0078	0,0031	C	Control-Region	-
P16468	16468	C	0,0016	0,0000	T	Control-Region	-
P16482	16482	G	0,0124	0,0170	A	Control-Region	-
P16491	16491	C	0,0016	0,0000	T	Control-Region	-
P16497	16497	G	0,0000	0,0015	A	Control-Region	-
P16508	16508	T	0,0016	0,0000	C	Control-Region	-
P16509	16509	C	0,0016	0,0000	T	Control-Region	-
P16519	16519	T	0,3354	0,3324	C	Control-Region	-
P16526	16526	A	0,0062	0,0046	G	Control-Region	-
P16527	16527	T	0,0031	0,0046	C	Control-Region	-
P16535	16535	A	0,0016	0,0000	G	Control-Region	-
