# Supplementary material for: The co-occurrence of mtDNA mutations on different oxidative phosphorylation subunits, not detected by haplogroup analysis, affects human longevity and is population specific
Source: Aging Cell. 2013 Dec 17;13(3):401–7. doi: 10.1111/acel.12186 (PMC4326891; doi:10.1111/acel.12186)
Supplement: Supplementary file 4 — Table S3. mtDNA mutations found in our study and previously associated with diseases. Table S4. Distribution of 90+ and controls from different centers participating in the GEHA project. Table S5. Sample distribution and general characteristics of the study participants. Table S6. Modified primer pairs for complete resequencing. [file acel0013-0401-sd4.pdf]

Table 3S. Rare mtDNA mutations associated with diseases and their distribution in the 90+ and controls of the present study.

		**Controls**		**90+**			
**Position**	Mutation type	N.	%	N.	%	Region	Disease
**827**	A/G	1	0,15	0	0,00	12S	Deafness
**961**	T/C	2	0,31	5	0,77	12S	Deafness
**1005**	T/C	1	0,15	0	0,00	12S	Deafness
**1438**	A/G	25	3,87	39	6,04	12S	Diabetes Mellitus
**1555**	A/G	0	0,00	3	0,46	12S	Deafness
**3196**	G/A	1	0,15	0	0,00	16S	Alzheimer & Parkinson Disease
**3308**	T/C	1	0,15	0	0,00	ND1	Encephalomyopathy, MELAS
**3460**	G/T	0	0,00	0	0,00	ND1	LHON
**3796**	A/G	3	0,46	5	0,77	ND1	Dystonia
**4295**	A/G	8	1,24	4	0,62	TI	Maternaly Inherited Hypertrophic Cardiomyopathy
**4336**	T/C	5	0,77	12	1,86	TQ	Alzheimer & Parkinson Disease<br>Deafness & Migraine
**5460**	G/A	36	5,57	31	4,80	ND2	Alzheimer & Parkinson Disease
**5628**	T/C	1	0,15	0	0,00	tRNA A	Mitochondrial Myopathy, CPEO MTTA CPEO
**5843**	A/G	1	0,15	0	0,00	TY	Mitochondrial Myopathy Cytopathy
**6489**	C/A	5	0,77	2	0,31	CO1	Epilepsy
**7444**	G/A	4	0,62	6	0,93	CO1	Deafness-Sensory Neural Hearing Loss
**8108**	A/G	0	0,00	1	0,15	CO1	Deafness-Sensory Neural Hearing Loss

**8302**	A/G	0	0,00	1	0,15	tRNA k	MTTK Encephalopathy
**8993**	T/C	0	0,00	0	0,00	ATP6	Leigh Syndrome
**10044**	A/G	8	1,24	3	0,46	TG	Encephalomyopathy Gastrointestinal Reflux and Sudden Infant Death Syndrome
**11084**	A/G	1	0,15	0	0,00	ND4	Encephalomyopathy, MELAS
**12026**	A/G	0	0,00	2	0,31	ND4	Diabetes Mellitus
**12192**	G/A	0	0,00	3	0,46	TH	Maternally Inherited Cardiomyopathy
**12308**	A/G	149	23,07	116	17,96	TL2	Mitochondrial Myopathy, CPEO, Stroke
**15497**	G/A	3	0,46	1	0,15	CYTB	MM, Execise Intolerance
**15498**	G/A	0	0,00	1	0,15	CYTB	Hypertrophic Cardiomyopathy
**15924**	A/G	17	2,63	27	4,18	TT	Mitochondrial Myopathy Lethal Infantile Mitochondrial Myopathy

Table 4S: Distribution of 90+ and controls from different centers participating to GEHA project.

Recruitment center	Country	No. 90+	No. younger controls
Belfast	Northern Ireland	64	64
Louvain	Belgium	80	88
Bologna	Italy	213	214
Kiel	Germany	94	96
Tampere	Finland	153	145
Montpellier	France	274	275
Newcastle	UK	99	100
Odense	Denmark	428	441
Leiden	Netherland	162	167
Warsaw	Poland	129	132
Rome	Italy	75	75
Sassari	Italy	47	52
Kiev	Ukraina	49	49
Calabria	Italy	125	152
Athens	Greece	94	103
**Total**		**2,086**	**2,153**

Table 5S: Sample distribution and general characteristics of the study participants.

	Males (N = 1,327)		Females (N = 2,912)	
	**90+ (%)**	**Controls (%)**	**90+ (%)**	**Controls (%)**
**N**	628 (47)	699 (53)	1,458 (50)	1,454 (50)
**Mean Age**	94,1 ± 2,5	61,7 ± 6,2	94,5 ± 2,6	61,7 ± 6,2
**Age range**	89-103	43-79	88-107	49-83

Table 6S: Modified primer pairs for the complete resequencing (M13 tag is not reported).

	**Forward**	**Reverse**
**Primer 41b**	5’- CTG CTT CTT CTC ACA TGA-3’	5’- TAG GTA GGA GTA GCG TG-3’
**Primer 43b**	5’- ATC AAT TGG CTT CCT AGG-3’	5’- GGC ATC CAT ATA GTC ACT-3’
**Primer 44b**	5’- CCT TAT CTG CTT CCT AGT-3’	5’-GGG TGC TAT AGG GTA AAT -3’
**Primer 45b**	5’- ATT TTG ACT ACC ACA ACT CA-3’	5’-ATA TGT GTT GGA GAT TGA GA -3’
